# Einbau von (Bioaktiven) Äquatorialen Liganden in Platin(IV)‐Komplexe

**DOI:** 10.1002/ange.202311468

**Published:** 2023-10-13

**Authors:** Alexander Kastner, Hemma Schueffl, Patrick A. Yassemipour, Bernhard K. Keppler, Petra Heffeter, Christian R. Kowol

**Affiliations:** ^1^ Universität Wien Fakultät für Chemie Institut für Anorganische Chemie Währinger Str. 42 1090 Wien Österreich; ^2^ Zentrum für Krebsforschung und Comprehensive Cancer Center Medizinische Universität Wien Borschkegasse 8a 1090 Wien Österreich; ^3^ Research Cluster “Translational Cancer Therapy Research” 1090 Wien Österreich; ^4^ Universität Wien Vienna Doctoral School in Chemistry (DoSChem) Währinger Str. 42 1090 Wien Österreich

**Keywords:** Bioanorganische Chemie, Krebsbekämpfung, Platin, Synthese Design

## Abstract

Platin(IV)‐Prodrugs sind aufgrund ihrer erhöhten Tumorselektivität und geringeren Nebenwirkungen äußerst interessante Alternativen zu Platin(II)‐Antitumortherapeutika. Im Gegensatz zur gängigen Theorie haben wir kürzlich beobachtet, dass äquatoriale Liganden von z. B. Oxaliplatin(IV)‐Komplexen unter Bildung von [(DACH)Pt(OH_eq_)_2_(OAc_ax_)_2_] hydrolysiert werden können. In der hier vorgestellten Arbeit untersuchten wir die Reaktivität und synthetische Verwendbarkeit dieses Komplexes, als Vorstufe für die Entwicklung neuartiger Platin(IV)‐Komplexe, welche mit herkömmlichen Methoden nicht zugänglich sind. Tatsächlich war es möglich die äquatorialen Hydroxidoliganden z. B. durch ein oder zwei monodentate Biotin‐Liganden, die unter Standardmethoden oxidiert werden würden, zu ersetzen. Die gebildeten Komplexe erwiesen sich als sehr stabil und zeigten auch nach der Reduktion eine langsame Ligandenfreisetzung, eine ideale Eigenschaft für lang zirkulierende zielgerichtete Strategien. Daraufhin wurden zwei Platin(IV)‐Komplexe mit äquatorialen Maleimiden, für die Bindung an Serumalbumin als natürlichen Nanocarrier, synthetisiert. Die Komplexe zeigten im Vergleich zu Oxaliplatin eine stark verlängerte Plasmahalbwertszeit und eine deutlich verbesserte Antitumoraktivität in vivo. Zusammenfassend ermöglicht diese neu entwickelte Syntheseplattform den einfachen und gezielten Einbau äquatorialer Liganden in Platin(IV)‐Komplexe. Des Weiteren können verschiedene (bioaktive) Einheiten koordiniert werden, wodurch sogar zielgerichtete dreifach‐wirksame Platin(IV)‐Prodrugs mit nur einem Platinzentrum möglich wären.

## Graphical abstract

Der direkte Einbau neuer äquatorialer Liganden in Platin(IV)‐Komplexe wurde erforscht, wodurch die Synthese sonst nicht verfügbarer Komplexe ermöglicht wurde. Koordination von Albumin‐bindenden Maleimidgruppen führte zu einer erhöhten Tumorakkumulation und Antitumoraktivität im Vergleich zu Oxaliplatin. Die vorgestellte Strategie erlaubt die Entwicklung von neuartigen zielgerichteten multifunktionalen Platin(IV)‐Komplexen.

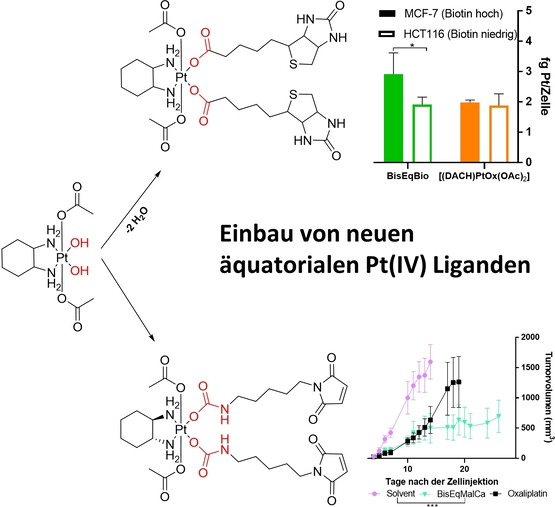

## Einleitung

Chemotherapie ist nach wie vor ein sehr wichtiger Bestandteil der Krebsbehandlung. Insbesondere Platin‐Komplexe werden häufig, auch in Kombination mit modernen Immuntherapeutika, eingesetzt.^[1]^ Bisher wurden weltweit nur drei Platinkomplexe für die klinische Anwendung zugelassen (Cisplatin, Carboplatin und Oxaliplatin), während fünf weitere ausschließlich in Asien angewendet werden.^[2]^ Ihr Wirkmechanismus basiert auf der Hydrolyse der labilen Liganden innerhalb der Zelle, gefolgt von der Anbindung an Guanin und Adenin der DNA, wodurch Apoptose ausgelöst wird.^[3]^ Diese Prozesse können jedoch auch in gesundem Gewebe auftreten und eine Vielzahl von dosislimitierenden Nebenwirkungen verursachen.^[4]^ Eine Möglichkeit diese Probleme zu reduzieren, besteht in der Verwendung von Platin(IV)‐Komplexen. Diese oxidierte Form ist kinetisch stabiler, weshalb viele Nebenreaktionen verhindert werden können. Darüber hinaus können die beiden eingeführten axialen Liganden für zielgerichtete Strategien, die Anbindung bioaktiver Liganden oder die Feinabstimmung physikochemischer Eigenschaften genutzt werden.^[5]^

Axiale und äquatoriale Liganden haben unterschiedliche Anforderungen: Axiale Liganden werden häufig zur Implementierung zusätzlicher Funktionalitäten in die Platin(IV)‐Komplexe eingesetzt und sollten daher hydrolysestabil sein. Im Gegensatz dazu sollten die äquatorialen “Abgangsgruppen“‐Liganden hydrolysieren, sobald der Komplex innerhalb der Krebszelle zu Platin(II) reduziert wird. Dennoch synthetisierten und verglichen einige Forschungsgruppen Komplexe, die z. B. Dichloracetat,^[6]^ Acetat^[7]^ oder Etacrynsäure,^[8]^ als axiale Liganden an Platin(IV) bzw. als äquatoriale Liganden an Platin(II) aufweisen. Diese Daten legen nahe, dass auch die Äquatorialebene für die direkte Anbindung bioaktiver Liganden genutzt werden kann.

Kürzlich berichteten wir über die unerwartete Hydrolyse der äquatorialen “Abgangsgruppe“ bestimmter Platin(IV)‐Komplexe unter physiologischen Bedingungen. Wir konnten zeigen, dass Oxali‐ und Satraplatin(IV)‐Komplexe schnell äquatorial hydrolysieren, während der Carboplatin(IV)‐Komplex völlig stabil war und das Cisplatin(IV)‐Analogon dazwischen lag. Dies ermöglichte auch die Synthese eines neuartigen Oxaliplatin(IV)‐Komplexes mit zwei äquatorialen Hydroxidoliganden: [(DACH)Pt(OH_eq_)_2_(OAc_ax_)_2_] (DACH=(1*R*,2*R*)‐1,2‐Diaminocyclohexan; Schema 1).^[9]^ Es stellte sich die Frage, ob dieser Komplex als synthetischer Vorläufer verwendet werden könnte, um die direkte und selektive Einführung neuer äquatorialer Liganden in Platin(IV)‐Komplexe zu ermöglichen. In der Literatur gibt es nur sehr wenige Veröffentlichungen zu Tetrahydroxidoplatin(IV)‐Komplexen, bei denen über solche Substitutionen berichtet wurde.^[10]^ Das Vorhandensein von vier Hydroxidogruppen, zwei axialen und zwei äquatorialen, ermöglicht jedoch keine spezifischen Reaktionen in der Äquatorialebene, was zu Isomerengemischen^[10c]^ oder der Koordination des gleichen Liganden in allen vier Positionen führt.^[10b]^

**Scheme 1:**
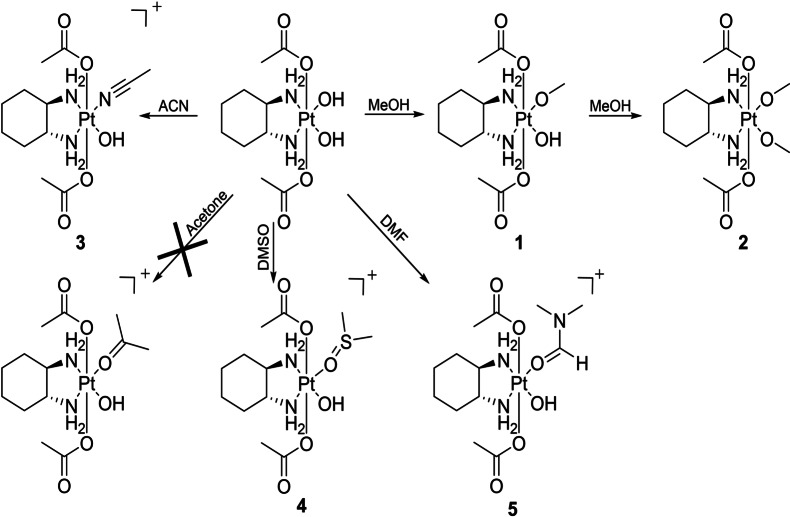
Übersicht über die Reaktionen von [(DACH)Pt(OH)_2_(OAc)_2_] mit verschiedenen Lösungsmitteln.

Üblicherweise beginnt die Herstellung von Platin(IV)‐Komplexen mit der Synthese des jeweiligen Platin(II)‐Kerns, der dann z. B. mit Wasserstoffperoxid oxidiert wird, um zwei axiale Hydroxido‐Gruppen einzuführen, die anschließend zu Estern, Carbonaten oder Carbamaten funktionalisiert werden können.^[11]^ Darüber hinaus wurde kürzlich über den Austausch axialer Halido‐Liganden berichtet, um beispielsweise N‐Heterocyclen einzuführen.^[12]^ Die selektive Substitution der äquatorialen “Abgangsgruppe” in Platin(IV)‐Komplexen wäre ein eleganter neuer Syntheseweg. Darüber hinaus ist der Oxidationsschritt nicht für jeden Liganden geeignet, da einige von ihnen selbst zur Oxidation neigen. Ein Beispiel dafür ist Biotin, dessen Thioether zu einem Sulfoxid oder sogar einem Sulfon oxidiert werden kann.^[13]^ Des Weiteren besitzen Schwefelatome eine hohe Affinität zum “weichen” Platin(II)‐Zentrum; dies wird durch die Einführung des Liganden in die “harte” Platin(IV)‐Oxidationsstufe verhindert. Biotin ist ein wichtiger Cofaktor in vielen Enzymen der Gluconeogenese oder Fettsäuresynthese.^[14]^ Es wird vom natriumabhängigen Multivitamin‐Transporter (SMVT) in die Zelle aufgenommen, der bei vielen Krebsarten überexprimiert ist, weshalb Biotin auch für die tumorspezifische Wirkstoffanreicherung genutzt wird.^[15]^ Biotin wurde bereits als axialer Ligand für Cisplatin(IV)‐Komplexe untersucht und zeigte vielversprechende Ergebnisse.^[16]^

Hier haben wir Biotin für die Etablierung der äquatorialen Substitutionsreaktion ausgewählt um die spezifischen Eigenschaften von [(DACH)Pt(OH_eq_)_2_(OAc_ax_)_2_] zu nutzen und Komplexe zu erzeugen, die auf herkömmlichen Synthesewegen nicht zugänglich sind. Anschließend wurde ihr hydrolytisches und reduktives Verhalten mit Analoga verglichen, welche Biotin in axialer Position tragen. Darüber hinaus wurde die biologische Aktivität gegen Krebszellen analysiert. Dies deutete darauf hin, dass die äquatoriale Substitution von Platin(IV)‐Komplexen zu stabilen Verbindungen führte, was solche Komplexe zu idealen Werkzeugen für Prodrugkonzepte macht. Das zeigte auch die Synthese der ersten äquatorialen Maleimid‐tragenden Platin(IV)‐Komplexe (die ebenfalls nicht mit Standardsynthesemethoden zugänglich sind) für Albumin‐vermittelten Transport.

## Ergebnisse und Diskussion

Im ersten Schritt wollten wir verstehen, ob die beiden äquatorialen Hydroxidogruppen von [(DACH)Pt(OH_eq_)_2_(OAc_ax_)_2_] für Substitutionsreaktionen geeignet sind und für neue Synthesestrategien verwendet werden können. Daher wurde [(DACH)PtOx(OAc)_2_] (Ox=Oxalat) in wässriger Lösung bei pH 9 für 24 Stunden gerührt, was zur Freisetzung des Oxalatliganden und zur Bildung von [(DACH)Pt(OH_eq_)_2_(OAc_ax_)_2_] führte.^[9]^ Anschließend wurde der isolierte Komplex 24 Stunden lang bei Raumtemperatur (RT) mit verschiedenen Lösungsmitteln (Aceton, Acetonitril (ACN), Dimethylformamid (DMF), Dimethylsulfoxid (DMSO), Methanol (MeOH)) inkubiert (Schema 1). Die Reaktionen wurden mittels Hochleistungsflüssigkeitschromatographie gekoppelt mit einem Massenspektrometer (HPLC‐MS) überwacht.

In MeOH bildete [(DACH)Pt(OH)_2_(OAc)_2_] die Mono‐ und Bis‐Methanolato‐Komplexe **1** und **2**. Dies steht im Einklang mit der Röntgenkristallstruktur von [(DACH)Pt(OH)_2_(OAc)_2_] aus MeOH/Diethylether, bei der ebenfalls beide Hydroxidoliganden durch MeOH ausgetauscht wurden.^[9]^ In ACN wurde ausschließlich der Mono‐ACN‐Komplex **3** gebildet. Im Gegensatz dazu koordinierte Aceton nicht an Platin, sondern es konnte aufgrund des ACN im HPLC‐Lösungsmittel wieder der Mono‐ACN‐Komplex **3** beobachtet werden (auch die direkte Injektion der Acetonlösung ins MS zeigte keine Koordination an Platin). Im Fall von DMSO und DMF wurden auch die jeweiligen Monolösungsmittelkomplexe **4** und **5** gebildet. Im Vergleich aller getesteten Lösungsmittel koordinierte MeOH am leichtesten und war das einzige Lösungsmittel, das zur Bildung einer Bis‐spezies führte.

Anschließend wurde die Stabilität der gebildeten Komplexe in wässriger Lösung bei physiologischem pH‐Wert untersucht. Nach 24‐stündiger Inkubation von 100 μM [(DACH)Pt(OH)_2_(OAc)_2_] in den verschiedenen Lösungsmitteln und Trocknen der Proben unter reduziertem Druck, wurden die verbleibenden Feststoffe in Phosphatpuffer bei pH 7.4 aufgenommen und mit HPLC‐MS auf ihre Stabilität gegen Hydrolyse getestet. Die Mono‐ACN‐ und ‐DMF‐Komplexe **3** und **5** hydrolysierten schnell und bildeten nach 1 Stunde erneut [(DACH)Pt(OH)_2_(OAc)_2_]. Die MeOH‐Komplexe **1** und **2** zeigten langsamere Hydrolyse ihrer Methoxidoliganden (Abbildung S2), mit einer Geschwindigkeit, die mit der Oxalatfreisetzung aus [(DACH)PtOx(OAc)_2_] vergleichbar ist.^[9]^ Die in DMSO gelöste Probe wurde im Laufe von 24 Stunden schwarz, weshalb die Hydrolysestabilität nicht getestet werden konnte. Eine genauere Untersuchung der HPLC‐MS‐Läufe ergab jedoch die Bildung des Tetraacetatokomplexes, der auch über präparative HPLC isoliert werden konnte (Daten nicht gezeigt). Dies legt nahe, dass axiale Acetatliganden, die während der Zersetzung/Reduktion freigesetzt wurden, an noch intakte Moleküle von [(DACH)Pt(OH)_2_(OAc)_2_] koordinieren (oder einen DMSO‐Liganden verdrängen) können. Folglich scheinen bereits recht geringe Mengen an Carbonsäuren (z. B. Acetat) ausreichend zu sein, um die Hydroxidoliganden in [(DACH)Pt(OH)_2_(OAc)_2_] zu ersetzen. Daher untersuchten wir die Reaktion von [(DACH)Pt(OH)_2_(OAc)_2_] mit 2–250 Äq. Essigsäure in Aceton (worin der Komplex stabil ist) für 24 h bei RT. Selbst mit nur 10 Äq. konnte im analytischen Maßstab eine vollständige Umwandlung zum Tetraacetatokomplex beobachtet werden. Daher wurde diese Reaktion hochskaliert und optimiert (Tabelle 1).

**Table 1:** 

Ligand	Temp	Solvent	[(DACH)Pt(OH)_2_(OAc)_2_] [mmol/l]	Ausbeute^[b]^
Essigsäure	RT^[a]^	Aceton	7.2	40 % Mono+ 20 % Bis
Essigsäure	RT	Wasser	7.2	keine Reaktion
Essigsäure	RT	DMF	7.2	40 % Mono+ 20 % Bis
Natriumoxalat	RT	DMF	7.2	10 % Mono+ 20 % Bis
Oxalsäure	RT	DMF	7.2	10 % Mono+ 30 % Bis
Biotin	RT	DMF	7.2	30 % Mono+ 0 % Bis
Biotin	RT	DMF	27	20 % Mono+ 20 % Bis
Biotin	50 °C	DMF	27	30 % Mono+ 60 % Bis

[a] RT=Raumtemperatur; [b] Ausbeute bestimmt mittels HPLC‐MS nach 24 h Reaktionszeit.

Alle Reaktionen wurden mit 5 mg [(DACH)Pt(OH)_2_(OAc)_2_], 10 Äq. des jeweiligen Liganden und einer Reaktionszeit von 24 h durchgeführt. Zunächst wurde die Reaktion mit Essigsäure in verschiedenen Lösungsmitteln getestet: In Wasser fand die Reaktion überhaupt nicht statt, was auch mit der hohen Stabilität von [(DACH)Pt(OH)_2_(OAc)_2_] in Medium und Serum übereinstimmt.^[9]^ Im Gegensatz dazu reagierten sowohl in Aceton als auch in DMF etwa 60 % des Dihydroxido‐Komplexes zu den Mono‐ oder Bis‐Acetat‐Spezies. Weitere Experimente wurden deshalb in dem vielseitigeren Lösungsmittel DMF durchgeführt. Anzumerken ist, dass die Verwendung von [(DACH)PtCl_2_(OAc)_2_] als Edukt unter den gleichen Bedingungen zu keiner Umwandlung führte. Als nächstes wurde untersucht ob [(DACH)Pt(OH)_2_(OAc)_2_] wieder in seinen ursprünglichen Oxaliplatin‐Kern umgewandelt werden kann. Sowohl die Zugabe von Natriumoxalat als auch Oxalsäure führte zum gewünschten Komplex (20 % bzw. 30 %). Dieser Vergleich zeigte außerdem, dass Säuren bei dieser Art von Reaktion bevorzugt sein könnten. Schließlich wurde der biologisch aktive Ligand Biotin verwendet. Während unter den “Standard“‐Bedingungen nur der Monokomplex gebildet wurde, konnte der Biskomplex durch eine 4‐fach konzentriertere Lösung und 50 °C Reaktionstemperatur in guter Ausbeute (60 %) erhalten werden. Unter diesen neuen Bedingungen bildeten sich die Mono‐ und Biskomplexe selbst mit nur einem (10 % Mono; 5 % Bis) oder zwei Äq. Biotin (5 % Mono; 15 % Bis). Bemerkenswert ist außerdem, dass bei Verwendung von 2.5 Äq. Biotin und 2.5 Äq. *O*‐(Benzotriazol‐1‐yl)‐*N*,*N*,*N*′,*N*′‐tetramethyluronium‐tetrafluoroborat (TBTU) als Kopplungsmittel ausschließlich der Biskomplex mit 30 % Ausbeute gebildet wurde. Bei nur begrenzt verfügbaren Mengen des Carboxylatliganden, könnte dies also eine interessante Alternativstrategie sein.

Zusätzlich untersuchten wir, ob ein äquatorialer Ligandenaustausch auch für einen Cisplatin‐Kern möglich ist, welcher im Allgemeinen viel resistenter gegen äquatoriale Hydrolyse ist.^[9]^ Daher erhöhten wir den pH‐Wert und die Temperatur weiter und inkubierten [(NH_3_)_2_PtCl_2_(OAc)_2_] in wässriger phosphatgepufferter Lösung bei pH 10 und 50 °C. Dies führte nach 24 h zu einer Mischung aus 80 % [(NH_3_)_2_Pt(OH_eq_)_2_(OAc_ax_)_2_] und 20 % [(NH_3_)_2_PtOH_eq_Cl_eq_(OAc_ax_)_2_]. Die erhaltene (OH_eq_)_2_‐Spezies wurde dann mit 10 Äq. Biotin bei 50 °C für 24 Stunden inkubiert, was zur ausschließlichen Bildung des Biotin/OH‐Komplexes führte (auch nach 48 Stunden Reaktionszeit). Bemerkenswert ist, dass bei der Inkubation der äquatorial gemischten OH/Cl‐Spezies [(NH_3_)_2_PtOH_eq_Cl_eq_(OAc_ax_)_2_] das OH mit der gleichen Geschwindigkeit wie bei der (OH_eq_)_2_‐Verbindung ausgetauscht wurde, was zum Biotin/Cl‐Komplex führte. Folglich können auch Cisplatin(IV)‐Spezies für äquatoriale Ligandensubstitutionsreaktionen verwendet werden.

Unser Fokus lag jedoch darauf, die Eigenschaften äquatorial substituierter Komplexe mit den jeweiligen axialen Gegenstücken zu vergleichen. Daher wurde das folgende Oxaliplatin(IV)‐Panel synthetisiert (Schema 2): der äquatoriale Mono‐Biotin‐ (**MonoEqBio**) und Bis‐Biotin‐Komplex (**BisEqBio**) und als axiale Referenzen die Oxaliplatin(IV)‐Komplexe mit einem (**MonoAxBio**) oder zwei Biotin‐Liganden (**BisAxBio**). Für die Aufreinigung von **MonoEqBio** auf der präparativen HPLC mussten reine Lösungsmittel verwendet werden, da die üblicherweise zugesetzten 0.1 % Ameisensäure oder Trifluoressigsäure (TFA) das OH am Platinkern ersetzten. Die Reinheit aller Komplexe wurde mittels NMR, MS und Elementaranalyse bestätigt. Versuche, die entsprechenden Platin(II)‐Biotin‐Referenzkomplexe zu synthetisieren, waren nicht erfolgreich. Höchstwahrscheinlich koordiniert das weiche Platin(II) leicht den Thioether von Biotin, was zu verschiedenen Spezies führt.

**Scheme 2:**
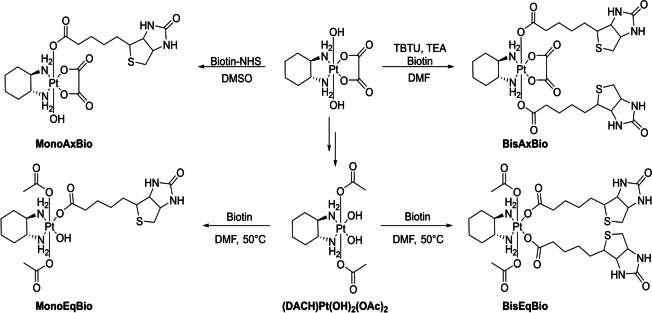
Strukturen der synthetisierten Biotin‐Platin(IV) Komplexe.

**Figure 1:**
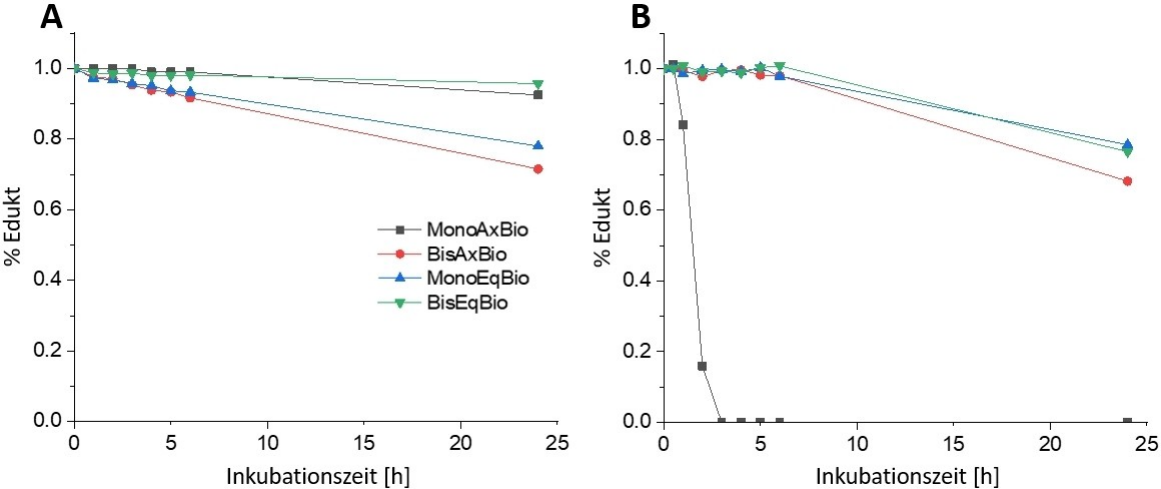
A) Äquatoriale Hydrolyse der untersuchten Platinkomplexe bei 37 °C (1 mM Komplex in 100 mM Phosphatpuffer pH 7.4). B) Reduktionskinetik der untersuchten Platinkomplexe bei 20 °C (1 mM Komplex mit 10 Äquivalenten Ascorbinsäure in 150 mM Phosphatpuffer pH 7.4).

Um die Eigenschaften der verschiedenen Oxaliplatin(IV)‐ähnlichen Biotinkomplexe zu vergleichen (Schema 2), wurde zunächst die Hydrolysestabilität der äquatorialen Liganden in den Platin(IV)‐Komplexen untersucht. Die Komplexe (1 mM) wurden in 100 mM Phosphatpuffer (pH 7.4) bei 37 °C inkubiert (Abbildung 1A). Während **MonoAxBio** recht stabil gegenüber äquatorialer Oxalathydrolyse war (8 % Mono‐OH‐Spezies nach 24 Stunden), zeigte **BisAxBio** nach 24 Stunden Inkubation erhebliche Anteile hydrolysierter Spezies (24 % Mono‐Hydroxido; 4 % Di‐Hydroxido). Die hohe Stabilität von **MonoAxBio** im Vergleich zu **BisAxBio** konnte durch die Referenzkomplexe [(DACH)PtOxOH_ax_OAc_ax_] und [(DACH)PtOx(OAc)_2_] bestätigt werden, die nach 24 Stunden eine äquatoriale Hydrolyse von 2 % bzw. ≈50 % am Platin(IV) Zentrum zeigten (Abbildung S3). Bei den Komplexen mit äquatorial gebundenem Biotin kehrte sich dieser Trend um: **BisEqBio** zeigte nur minimale Hydrolyse (4 % nach 24 h), während 22 % von **MonoEqBio** nach 24 h zur Dihydroxido‐Spezies hydrolysiert waren, was auch den Nachweis von freigesetztem Biotin ermöglichte (Abbildung S4). Diese Daten zeigen, dass im Hinblick auf die äquatoriale Hydrolyse von Platin(IV)‐Komplexen die beiden direkt benachbarten Carbonsäuregruppen im Oxalatliganden von **BisAxBio**, verglichen mit den beiden einzähnigen Carboxylatliganden in **BisEqBio**, eine deutlich geringere Koordinationsstärke aufweisen. Dementsprechend war auch das Carboplatin‐Analogon [(DACH)Pt(CBDCA)(OAc)_2_] (CBDCA=1,1‐Cyclobutandicarbonsäure) mit einem Kohlenstoffatom zwischen den zweizähnigen Carboxylaten sehr stabil (2 % Hydrolyse nach 24 h; Daten nicht gezeigt), was darauf hindeutet, dass sich **BisEqBio** eher wie ein Carboplatin‐ als ein Oxaliplatin‐Derivat verhält.

Die Reduktionskinetik der Komplexe in Gegenwart von 10 Äq. Ascorbinsäure bei 20 °C (Abbildung 1B) ergab, dass **MonoAxBio** nach 3 Stunden vollständig reduziert war, was im Einklang mit der Literatur zu anderen Platin(IV)‐Komplexen mit einer axialen OH‐Gruppe ist.^[17]^ Die anderen drei Komplexe waren viel stabiler, wobei **MonoEqBio** und **BisEqBio** nach 24 Stunden noch zu etwa 80 % und **BisAxBio** zu etwa 70 % intakt waren (wiederum konnte für die axialen Biotinkomplexe **MonoAxBio** und **BisAxBio** die Freisetzung von Biotin und Oxaliplatin bestätigt werden; Abbildung S5). Interessanterweise beschleunigte die freie OH‐Einheit in äquatorialer Position (**MonoEqBio**) die Reduktion nicht, wie dies für das axiale Analog **MonoAxBio** beobachtet wurde.

**Figure 2:**
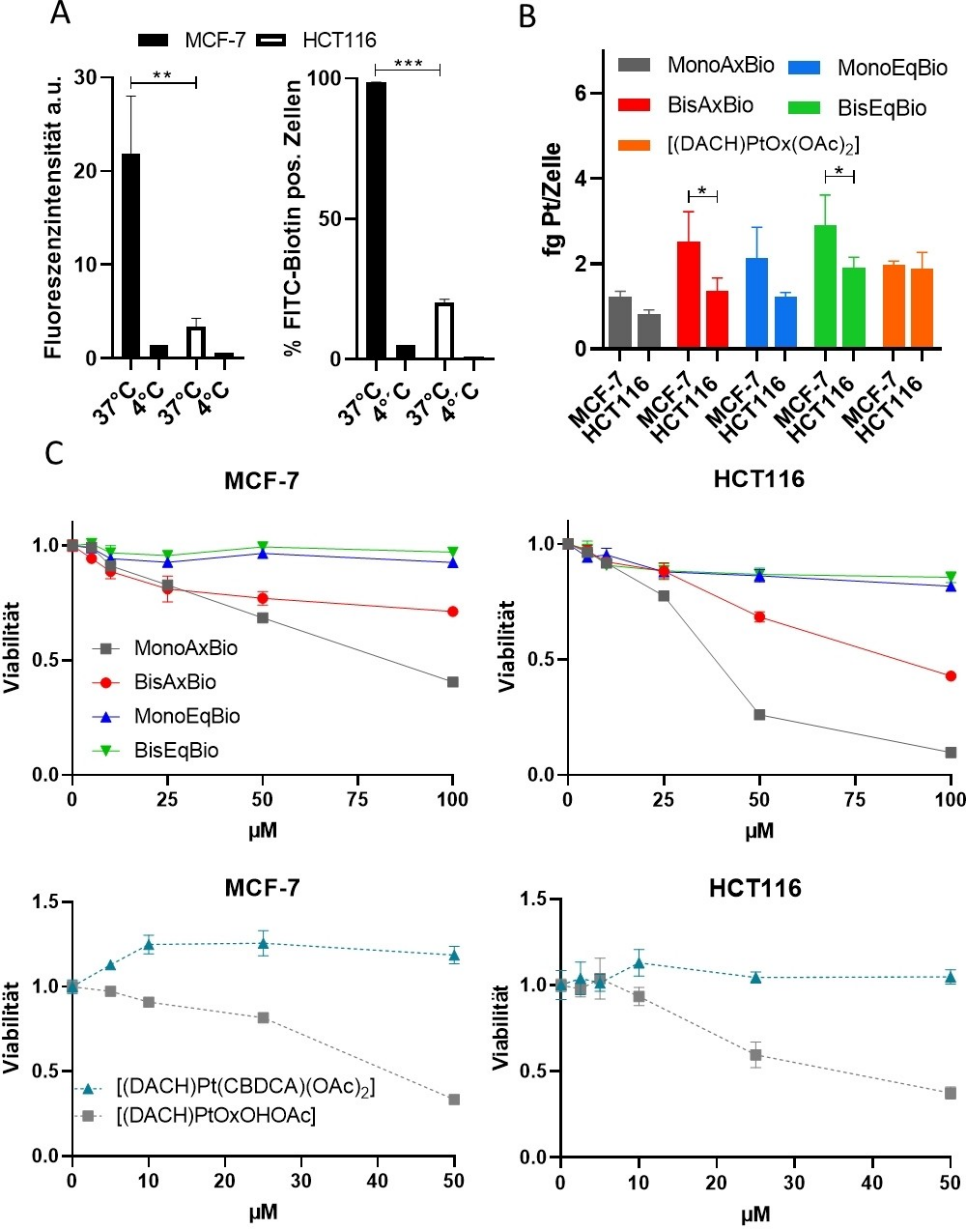
Charakterisierung der zellulären Biotin‐ und Platinkomplexaufnahme sowie der Antitumoraktivität der Platin(IV)‐Komplexe in MCF‐7‐ (viel SMVT) und HCT116‐Zellen (wenig SMVT). A) Die Zellen wurden mit FITC‐markiertem Biotin (20 μM) bei 37 °C oder 4 °C für 5 Stunden inkubiert. Die mittlere FITC‐Fluoreszenzintensität (links) und FITC‐positive Zellen (rechts) wurden durch Durchflusszytometrie bestimmt (ex/em: 488/530 nm, die Fluoreszenzintensität wurde auf die automatische Fluoreszenzkontrolle normalisiert). B) Die zelluläre Aufnahme der angegebenen Verbindungen (10 μM) wurde durch ICP‐MS nach 3‐stündiger Inkubation bestimmt. Die Werte in (A) und (B) wurden als Mittelwerte±SD von drei unabhängigen Experimenten dargestellt. Die statistische Signifikanz wurde durch einfaktorielle ANOVA und Dunnetts Mehrfachvergleichstest getestet (*p<0.05, **p<0.01 und ***p<0.001). (C) Die Antitumoraktivität von Biotin‐haltigen Platinverbindungen und Platin‐Referenzverbindungen nach 72 Stunden wurde mittels MTT‐basierter Tests bestimmt. Im Fall von (C) und (D) beziehen sich die Werte auf Mittelwerte ±SD eines repräsentativen Experiments, das dreifach durchgeführt wurde.

Um den Einfluss der äquatorialen/axialen Ligandenkoordination auf die zelluläre Aufnahme zu testen, wurden zwei Krebszellmodelle mit unterschiedlicher zellulärer SMVT‐Expression, was die Fähigkeit zur Biotinaufnahme wiederspiegelt, eingesetzt (humaner Darmkrebs HCT116 und humaner Brustkrebs MCF‐7).^[18]^ Die erhöhte und aktive Biotinaufnahme von MCF‐7‐Zellen (im Vergleich zu HCT116‐Zellen) wurde zuerst mittels Durchflusszytometrie nach Inkubation mit FITC‐markiertem Biotin bestätigt (Abbildung 2A). Anschließend wurde Massenspektrometrie mit induktiv gekoppeltem Plasma (ICP‐MS) verwendet, um die intrazellulären Platinlevels nach der Behandlung mit den neuen Biotin‐Platin(IV)‐Derivaten zu messen. [(DACH)PtOx(OAc)_2_] wurde dabei als Referenz verwendet. Wie in Abbildung 2B gezeigt, hatten die SMVT‐hohen MCF‐7‐Zellen deutlich höhere Platinniveaus als SMVT‐niedrige HCT116‐Zellen, insbesondere im Fall der beiden Bis‐Biotin‐Verbindungen. Ein ähnliches Muster wurde für die Monobiotin‐Verbindungen beobachtet (auch wenn es keine statistische Signifikanz erreichte). Im Gegensatz dazu wurde die Aufnahme von [(DACH)PtOx(OAc)_2_] nicht durch die SMVT‐Expression beeinflusst.

Um die Antitumoraktivität der Komplexe zu testen, verglichen wir zunächst die Wirkung von Oxaliplatin und [(DACH)PtOx(OAc)_2_] in MCF‐7 und HCT116 Zellen nach 72 Stunden mittels MTT‐basierter Tests (Abbildung S6). Oxaliplatin war deutlich aktiver in HCT116 (IC_50_ von 1.88 μM) als in MCF‐7‐Zellen (IC_50_ >10 μM). Dies steht in guter Übereinstimmung damit, dass Oxaliplatin die Standardtherapie gegen fortgeschrittenen Darmkrebs ist.^[19]^ Auch im Fall von [(DACH)PtOx(OAc)_2_] war die Antitumoraktivität gegen HCT116 Zellen stärker ausgeprägt als gegen MCF‐7‐Zellen (16.8 μM bzw. 34.4 μM), im Allgemeinen jedoch aufgrund der Prodrug‐Eigenschaften des Platin(IV)‐Komplexes deutlich geringer.^[20]^ Die vier Biotin‐konjugierten Platin(IV)‐Verbindungen (Schema 2) zeigten unter den gleichen Bedingungen starke Unterschiede (Abbildung 2C). Die beiden äquatorialen Biotin‐konjugierten Substanzen zeigten bis zur höchsten Konzentration von 100 μM keine Aktivität, **BisAxBio** war leicht zytotoxisch und **MonoAxBio** war das aktivste Derivat. Insgesamt waren das allgemeine Muster und die Aktivität der getesteten Substanzen in beiden Zellmodellen ähnlich (besonders interessant angesichts der verringerten Empfindlichkeit von MCF‐7 gegenüber Oxaliplatin), was darauf hindeutet, dass die Unterschiede in der Antitumoraktivität nicht durch die unterschiedliche Effizienz der Biotinaufnahme erklärt werden können. Die höhere Aktivität von **MonoAxBio** lässt sich durch die, im Vergleich zu den anderen Derivaten, sehr schnelle Reduktion erklären (Abbildung 1B). Dementsprechend zeigten Zellviabilitätsexperimente mit dem Referenzkomplex [(DACH)PtOxOHOAc] eine ähnliche Antitumoraktivität (Abbildung 2D). Es war jedoch unerwartet, dass **MonoEqBio** und **BisEqBio** mit einzähnigen Biotinliganden noch weniger aktiv waren als **BisAxBio** mit einem zweizähnigen Oxalatliganden. Da die Reduktionseigenschaften dieser drei Komplexe recht ähnlich sind (Abbildung 1B), vermuteten wir, dass der Grund in der Hydrolysegeschwindigkeit der gebildeten Platin(II)‐Komplexe liegen muss. Tatsächlich zeigten anschließend durchgeführte Zellviabilitätsexperimente mit der Carboplatin‐Referenz [(DACH)Pt(CBDCA)(OAc)_2_] eine mit den äquatorialen Biotin‐Komplexen vergleichbare Aktivität (Abbildung 2D). Dies legt nahe, dass die spezifischen Reduktions‐ und Hydrolyseeigenschaften der einzelnen Derivate entscheidend für ihre Antitumoraktivitäten sind.

Bemerkenswert ist, dass eine längere Inkubation (10‐tägige Koloniebildungstests) nicht nur die allgemeinen Aktivitäten der Verbindungen steigerte (aufgrund der längeren Zeit für die Prodrug‐ und Platin(II)‐Aktivierung), sondern auch das Reaktionsmuster der äquatorialen Komplexe umkehrte (Abbildung S7). Somit waren die Komplexe mit äquatorialem Biotin nun wirksamer gegen die oxaliplatin‐resistenten SMVT‐hohen MCF‐7 als gegen HCT116‐Zellen. Im Gegensatz dazu zeigten die axial konjugierten Biotin‐Verbindungen ein Aktivitätsmuster, das mit den 72‐Stunden‐Experimenten vergleichbar war. Dies deutet darauf hin, dass die genaue Position der Liganden die Aktivität sowie das (Platin−)Resistenzmuster der Komplexe deutlich beeinflussen kann.

Zusammenfassend ist klar ersichtlich, dass **MonoEqBio** und **BisEqBio** hochstabile Platin(IV)‐Komplexe sind, die nach der Reduktion Platin(II)‐Derivate mit langsamer Ligandenfreisetzung erzeugen, die eher mit Carboplatin als mit Oxaliplatin vergleichbar sind. Daher stellten wir die Hypothese auf, dass diese Eigenschaften äquatorialer einzähniger Carboxylatliganden ideal für die Verwendung lang zirkulierender zielgerichteter Systeme sind, bei denen eine langsame Freisetzung/Aktivierung erwünscht ist. Miriplatin‐Emulsionen^[21]^ (in Japan gegen hepatozelluläres Karzinom zugelassen) oder liposomales Aroplatin^[22]^ (bis zur klinischen Phase II untersucht) sind Vertreter dieser Art von Platin(II)‐Komplexen mit zwei einzähnigen Carboxylatliganden. Eine elegante zielgerichtete Strategie ist die Verwendung von Albumin als Nanocarrier mittels Maleimidchemie. Erst kürzlich berichteten wir über die Entwicklung von Platin(IV)‐Komplexen mit axialen Maleimidliganden.[^20b^, ^23^] Allerdings würde die Einführung des albuminbindenden Maleimids an äquatorialer Position die Bindung von zwei (verschiedenen) synergistischen Therapeutika an den freien axialen Positionen ermöglichen. Dadurch wären zielgerichtete dreifach‐wirksame Prodrugs innerhalb eines Platinkomplexes erreichbar.

**Figure 3:**
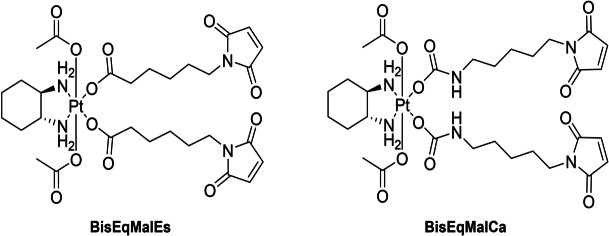
Strukturen synthetisierter maleimidhaltiger Platin(IV)‐Komplexe.

Daher synthetisierten wir zwei verschiedene äquatoriale Maleimid‐Platin(IV)‐Komplexe (Abbildung 3) nach dem oben beschriebenen Verfahren. **BisEqMalEs** wurde durch Inkubation von 6‐Maleimidhexansäure und [(DACH)Pt(OH)_2_(OAc)_2_] synthetisiert, während für das Carbamatanalogon (**BisEqMalCa**) Maleimidisocyanat verwendet wurde. Zu bemerken ist, dass solche maleimidhaltigen Komplexe nicht mit herkömmlichen Synthesemethoden aus den jeweiligen Platin(II)‐Vorläufern synthetisiert werden könnten, da bereits die Oxidation mit H_2_O_2_ zur Hydrolyse des Maleimids führt. Unserem Wissen nach ist **BisEqMalCa** außerdem der erste Platin(IV)‐Komplex mit äquatorialen Carbamat‐Abgangsgruppen. Die Inkubation der Komplexe bei pH 7.4 ergab eine hohe Stabilität (Abbildung S8), mit Ausnahme der bekannten Maleimidhydrolyse bei physiologischem pH (Abbildung S9).^[24]^ Dieses Phänomen erschwerte auch eine quantitative Bewertung der Reduktionsrate, da sich beide Prozesse überlagerten; trotzdem konnten für **BisEqMalEs** und **BisEqMalCa** ähnliche Reduktionsraten beobachtet werden (Abbildung S10). Darüber hinaus zeigten Albuminbindungsstudien mittels Größenausschlusschromatographie (SEC) gekoppelt mit ICP‐MS schnelle Bindungsraten für beide Komplexe in fetalem Kälberserum (FCS, gepuffert mit 150 mM Phosphatpuffer, um einen stabilen pH‐Wert zu gewährleisten) bei 37 °C (Abbildung S11).

**Figure 4:**
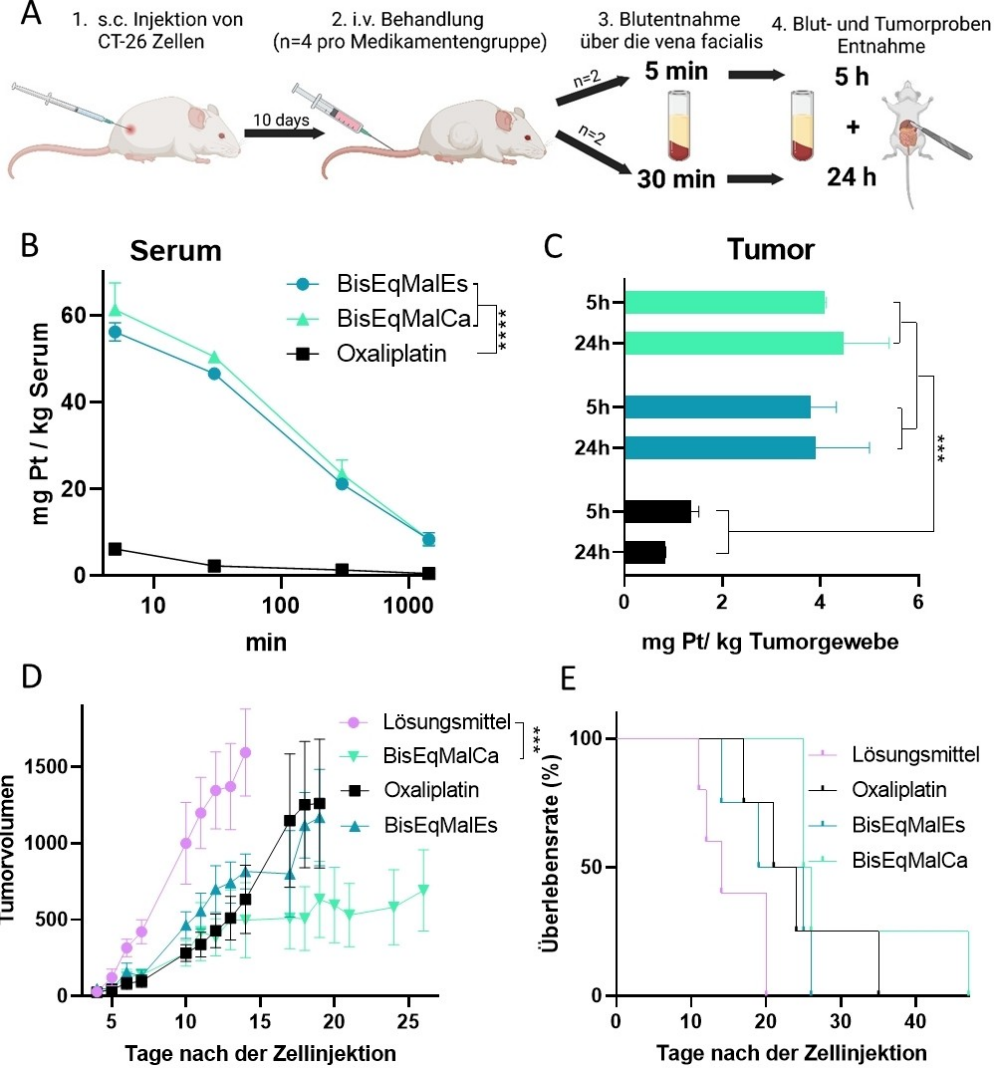
Pharmakologische Bewertung und Antitumoraktivität der Platinkomplexe in CT‐26‐tragenden Balb/c‐Mäusen. Für die pharmakologischen Studien wurden die Tiere einmal mit einer Dosierung äquimolar zu 9 mg/kg Oxaliplatin i. v. behandelt. A) Behandlungs‐ und Probenentnahmeschema für pharmakologische Studien. B) Serumproben wurden nach 5 Minuten, 30 Minuten, 300 Minuten und 1440 Minuten gesammelt; C) Tumorproben wurden nach 5 und 24 Stunden entnommen. Der Platingehalt aller Proben wurde mittels ICP‐MS gemessen. Für die Bestimmung der Antitumoraktivität wurden CT‐26‐tragende Balb/c‐Mäuse zwei Wochen lang zweimal pro Woche mit einer Dosierung äquimolar zu 9 mg/kg Oxaliplatin i. v. behandelt. (D) Einfluss auf das Tumorwachstum; die Daten werden als Mittelwert±SEM dargestellt. (E) Das Gesamtüberleben wurde über eine Kaplan‐Meier‐Kurve dargestellt. Die statistische Signifikanz für (B) und (D) wurde durch eine Zwei‐Wege‐ANOVA getestet (***p<0.001). Die Oxaliplatin‐Daten für die pharmakologische Analyse wurden von Schueffl et al. verwendet.^[20b]^

Die Beurteilung von Maleimidkomplexen in Zellkulturen ist aufgrund der Reaktion mit dem artifiziell hohen Gehalt an Aminosäuren im Kulturmedium meist problematisch. Folglich wurden die beiden neuen Albumin‐bindenden Verbindungen direkt in vivo an CT‐26‐Darmkrebs tragenden Balb/c‐Mäusen auf ihre pharmakokinetischen Eigenschaften getestet (Abbildung 4A). Tatsächlich führte die Maleimid‐Funktionalisierung nicht nur zu einer deutlich verlängerten Plasmahalbwertszeit, sondern auch zu einer erhöhten Akkumulation im bösartigen Gewebe im Vergleich zu freiem Oxaliplatin (Abbildung 4B&C), was gut mit früheren Erkenntnissen über axial maleimidhaltige Platin(IV)‐Derivate übereinstimmt.[^20b^, ^25^] Dies führte auch zu einer überlegenen Antitumoraktivität von **BisEqMalCa** im Vergleich zu Oxaliplatin (Abbildung 4D), während das bei **BisEqMalEs** nicht der Fall war. Erst vor kurzem wurde ebenfalls eine deutlich höhere Aktivität des axial “Carbamat‐verknüpften“ Maleimids im Vergleich zum ”Ester‐verknüpften“ Analogon für Platin(IV)‐Verbindungen beobachtet,^[25]^ obwohl die Gründe dafür bisher noch nicht bekannt sind.

## Zusammenfassung

Alles in allem stellt diese Arbeit eine neue Strategie für die Synthese von Platin(IV)‐Komplexen vor, mit der Einführung der äquatorialen Liganden als letzten Schritt. Dies ermöglicht insbesondere den Einbau empfindlicher Liganden, die die Platin(II)‐Oxidation mit H_2_O_2_ nicht überstehen würden. Die äquatorialen Biotinliganden der neu synthetisierten Komplexe **MonoEqBio** und **BisEqBio** zeigten eine sehr langsame Hydrolysekinetik (vergleichbar mit Carboplatin), ideal für zielgerichtete Strategien mit langer Zirkulation. Tatsächlich zeigten zwei Komplexe mit äquatorial eingeführten Maleimidliganden nach Bindung an endogenes Albumin (**BisEqMalEs** und **BisEqMalCa**) deutlich verbesserte pharmakokinetische Eigenschaften und Tumorakkumulation bei Mäusen. **BisEqMalCa** zeigte außerdem eine deutlich höhere Antitumoraktivität im Vergleich zur zugelassenen Referenz Oxaliplatin. Durch die Verschiebung der zielgerichteten Einheit von der typischen axialen Position in die Äquatorialebene werden zwei freie axiale Positionen für die Koordination (verschiedener) synergistischer Wirkstoffe generiert, was die Entwicklung neuer Arten von Platin(IV)‐Prodrugs mit dreifacher Wirkung ermöglicht.

## Interessenkonflikt

Die Autoren erklären, dass keine Interessenkonflikte vorliegen.

1

## Supporting information

As a service to our authors and readers, this journal provides supporting information supplied by the authors. Such materials are peer reviewed and may be re‐organized for online delivery, but are not copy‐edited or typeset. Technical support issues arising from supporting information (other than missing files) should be addressed to the authors.

Supporting Information

## Data Availability

Die Daten, die die Ergebnisse dieser Studie unterstützen, sind auf begründete Anfrage beim Autor erhältlich.
