# Supplementary material for: Einbau von (Bioaktiven) Äquatorialen Liganden in Platin(IV)‐Komplexe
Source: Angew Chem Weinheim Bergstr Ger. 2023 Oct 13;135(46):e202311468. doi: 10.1002/ange.202311468 (PMC10952677; doi:10.1002/ange.202311468)
Supplement: Supplementary file 1 — Supporting Information [file ANGE-135-0-s001.pdf]

Angewandte Chemie
Eine Zeitschrift der Gesellschaft Deutscher Chemiker

Supporting Information

Einbau von (Bioaktiven) Äquatorialen Liganden in Platin(IV)-Komplexe

A. Kastner, H. Schueffl, P. A. Yassemipour, B. K. Keppler, P. Heffeter, C. R. Kowol**

Chemicals and instrumentation

Potassium tetrachloridoplatinate ($K_2[PtCl_4]$) was purchased from Johnson Matthey (Switzerland). Water for synthesis was taken from a reverse osmosis system. For HPLC measurements Milli-Q water (18.2 MΩ·cm, Merck Milli-Q Advantage, Darmstadt, Germany) was used. Other chemicals and solvents were purchased from commercial suppliers (Sigma Aldrich, Merck and Fisher Scientific). Electrospray ionization (ESI) mass spectra were recorded on a Bruker amaZon SL ion trap mass spectrometer in positive and/or negative mode by direct infusion. High-resolution mass spectra were measured on a Bruker maXis™ UHR ESI time of flight mass spectrometer. One- and two-dimensional ^{1}H-NMR and ^{13}C-NMR spectra were recorded on a Bruker Avance III 500 or AV III 600 spectrometer at 298 K. For NMR spectra the solvent residual peak was taken as internal reference. For NMR numbering of the compounds, see Figure S1. NMR spectra are provided as Figures S12 – S23. Elemental analysis measurements were performed on a Perkin Elmer 2400 CHN Elemental Analyzer at the Microanalytical Laboratory of the University of Vienna. Compounds were purified by preparative RP-HPLC using a Waters XBridge C18 column on an Agilent 1200 Series system. Milli-Q water and acetonitrile were used as eluents and a flow rate of 17 ml/min.

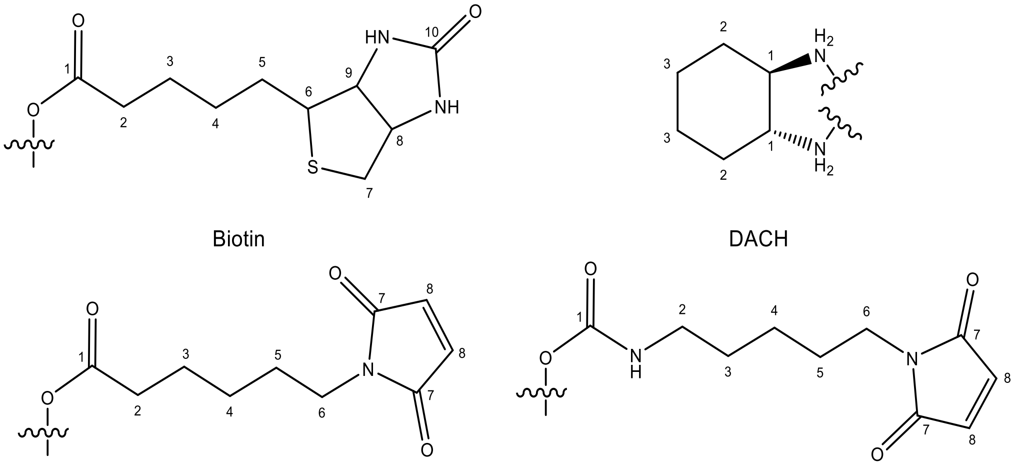

Figure S1. NMR numbering scheme for the used ligands

Synthesis of complexes

The complexes $[(DACH)PtOx(OH)_2]$[1], $[(DACH)PtOx(OAc)_2]$[2], $[(DACH)Pt(OH_{eq})_2(OAc_{ax})_2]$[2], $[(DACH)Pt(CBDCA)(OAc)_2]$[3] and $[(DACH)PtOxOH_{ax}OAc_{ax}]$[4], as well as the biotin-NHS-ester[5] and maleimide isocyanate[6] were synthesized according to literature. For the equatorial biotin complexes $[(DACH)Pt(OH)_2OAc_2]$ was used without prior purification (in the presence of phosphate buffer salts used

for oxalate hydrolysis). For the complexes bearing an equatorial maleimide [(DACH)Pt(OH)$_2$OAc$_2$] was purified before use with preparative HPLC using an Atlantis T3 Prep column, H_2O/ACN (without the addition of TFA) as solvents and a gradient of 1-15 % ACN in 15 minutes.

MonoAxBio

(OC-6-33)-[(1*R*,2*R*)-cyclohexane-1,2-diamine]hydroxido-oxalato[5-(2-oxohexahydro-1*H*-thieno[3,4-d]imidazol-4-yl)pentanoato]platinum(IV)

The synthesis was carried out based on a procedure of Wang and coworkers for cisplatin(IV)-biotin complexes.[5] [(DACH)PtOx(OH)$_2$] (50 mg, 0.133 mmol) was suspended in 3 ml dry DMSO and the Biotin-NHS-ester (46 mg, 0.133 mmol) was added. The reaction was stirred under argon atmosphere at 60°C overnight. Afterwards, the solvent was removed in vacuo and the crude product was purified via preparative RP-HPLC with an isocratic gradient of 13% ACN. Yield: 50 mg (65%) as a white solid. ^{1}H-NMR (500 MHz, DMSO-d_6): δ = 8.60 (m, 1H, NH_2), 8.20 (b, 1H, NH_2), 7.81 (b, 1H, NH_2), 7.12 (m, 1H, NH_2), 6.37 (b, 2H, N*H*), 4.31 (t, 1H, Biotin-8, *J* = 6.27 Hz), 4.13 (m, 1H, Biotin-9), 3.08 (b, 1H, Biotin-6), 2.82 (dd, 1H, Biotin-7, *J* = 12.44 Hz, *J* = 4.45 Hz), 2.57 (d, 2H, Biotin-7, *J* = 11.99 Hz), 2.53+2.47(under the solvent peak, 2H, DACH-1), 2.19 (t, 2H, Biotin-2, *J* = 7.27 Hz), 2.07 (dd, 2H, DACH-2, *J* = 27.79 Hz, *J* = 11.81 Hz), 1.58 (m, 1H, Biotin-4), 1.54-1.38 (m, 6H, DACH-3,DACH-2,Biotin-3,Biotin-4), 1.38-1.21 (m, 3H, DACH-2,Biotin-5), 1.11 (m, 2H, DACH-3) ppm. ^{13}C-NMR (125 MHz, DMSO-d_6): δ = 182.21 (Biotin-1), 163.81 (Oxalate), 163.80 (Oxalate), 162.75 (Biotin-10), 61.56 (DACH-1), 61.01 (Biotin-9), 60.11 (DACH-1), 59.22 (Biotin-8), 55.43 (Biotin-6), 39.85 (under the solvent peak, Biotin-7), 36.58 (Biotin-2), 30.88 (DACH-2), 30.77 (DACH-2), 28.17 (Biotin-5), 28.03 (Biotin-4), 25.57 (Biotin-3), 23.68 (DACH-3), 23.64 (DACH-3) ppm; MS: calcd. for $[C_{18}H_{30}N_4O_8PtS-H^+]^+$: 658.1506, found: 658.1507; elemental analysis calcd. for $C_{18}H_{30}N_4O_8PtS \cdot 1$ TFA $\cdot$ 0.5 H_2O: C: 30.77, H: 4.13, N: 7.18, S: 4.11, found: C: 30.68, H: 4.13, N: 7.33, S: 4.27.

BisAxBio

(OC-6-33)-[(1*R*,2*R*)-cyclohexane-1,2-diamine]oxalatodi[5-(2-oxohexahydro-1*H*-thieno[3,4-d]imidazol-4-yl)pentanoato]platinum(IV)

The synthesis was carried out based on a procedure of Wang and coworkers for cisplatin(IV)-biotin complexes.[5] Biotin (50 mg, 0.203 mmol) was dissolved in 3 ml dry DMF. TBTU (65.5 mg, 0.203 mmol) and TEA (20 mg, 0.203 mmol) were added and the mixture was stirred under Ar. After 30 minutes [(DACH)PtOx(OH)$_2$] (35 mg, 0.081 mmol) was added and the mixture was stirred for another 48 h. Afterwards, the solvent was removed in vacuo and the compound was purified via preparative RP-HPLC

with an isocratic gradient of 20% ACN. Yield: 48 mg (67%) as a white solid. ^{1}H-NMR (500 MHz, DMSO-d_6): δ = 8.36 (m, 4H, N*H*$_2$), 6.36 (b, 4H, N*H*), 4.30 (dd, 2H, Biotin-8, *J* = 7.45 Hz, *J* = 4.90 Hz), 4.12 (dd, 2H, Biotin-9, *J* = 7.72 Hz, *J* = 4.45 Hz), 3.07 (m, 2H, Biotin-6), 2.81 (dd, 2H, Biotin-7, *J* = 12.26 Hz, *J* = 4.99 Hz), 2.57 (d, 2H, Biotin-7, *J* = 12.71 Hz), 2.52 (under the solvent peak, 2H, DACH-1), 2.26 (t, 4H, Biotin-2, *J* = 7.36 Hz), 2.13 (d, 2H, DACH-2, *J* = 11.99 Hz), 1.58 (m, 2H, Biotin-5), 1.53-1.37 (m, 10H, DACH-2,DACH-3,Biotin-3,Biotin-5), 1.29 (m, 4H, Biotin-4), 1.14 (m, 2H, DACH-3) ppm. ^{13}C-NMR (125 Mhz, DMSO-d_6): δ = 181.03 (Biotin-1), 163.35 (Oxalate), 162.71 (Biotin-10), 61.19 (Biotin-9), 60.99 (DACH-1), 59.20 (Biotin-8), 55.39 (Biotin-6), 39.75 (under the solvent peak, Biotin-7), 35.65 (Biotin-2), 30.96 (DACH-2), 28.06 (Biotin-5), 28.00 (Biotin-4), 25.35 (Biotin-3), 23.53 (DACH-3) ppm; MS: calcd. for $[C_{28}H_{44}N_6O_{10}PtS_2\text{-}H^+]^+$: 884.2283, found: 884.2282; elemental analysis calcd. for $C_{28}H_{44}N_6O_{10}PtS_2$ · 1 TFA: C: 36.11, H: 4.55, N: 8.42, S: 6.43, found: C: 36.15, H: 4.82, N: 8.70, S: 6.74.

MonoEqBio and BisEqBio

(OC-6-32)-Diacetato[(1*R*,2*R*)-cyclohexane-1,2-diamine]hydroxido[5-(2-oxohexahydro-1*H*-thieno[3,4-d]imidazol-4-yl)pentanoato]platinum(IV)

and

(OC-6-33)-Diacetato[(1*R*,2*R*)-cyclohexane-1,2-diamine]di[5-(2-oxohexahydro-1*H*-thieno[3,4-d]imidazol-4-yl)pentanoato]platinum(IV)

Crude $[(DACH)Pt(OH)_2(OAc)_2]$ (125 mg Pt complex, 0.271 mmol) was suspended in 10 ml DMF, biotin (661 mg, 2.71 mmol) was added and the mixture was stirred for 24 h at 50°C. The solvent was removed in vacuo and the crude product was purified via preparative RP-HPLC with a gradient of 15-40% ACN (solvents without added TFA). Yield: 81 mg (43%) as well as 122 mg BisEqBio (49%), both as a white solid.

<u>Characterization of MonoEqBio:</u>

^{1}H-NMR (500 MHz, DMSO-d_6): δ = 8.72 (b, 1H, N*H*$_2$), 8.45 (t, 1H, N*H*$_2$, *J* = 10.54 Hz), 8.16 (t, 1H, N*H*$_2$, *J* = 9.99 Hz), 7.67 (b, 1H, N*H*$_2$), 5.82 (b, 2H, N*H*), 4.54 (t, 1H, Biotin-8, *J* = 6.64 Hz), 4.15 (m, 1H, Biotin-9), 3.19 (m, 1H, Biotin-6), 2.94 (d, 1H, Biotin-7, *J* = 11.44 Hz), 2.86 (m, 1H, Biotin-7), 2.57 (m, 2H, DACH-1), 2.18 (b, 2H, DACH-2), 2.13 (t, 2H, Biotin-2, *J* = 8.45 Hz), 1.87 (s, 3H, Acetate), 1.86 (s, 3H, Acetate), 1.73-1.61 (m, 3H, Biotin-3,Biotin-5), 1.57-1.45 (m, 4H, DACH-3,Biotin-4,Biotin-5), 1.37 (m, 2H, DACH-2), 1.24 (m, 1H, Biotin-4), 1.09 (m, 2H, DACH-3) ppm. ^{13}C-NMR (125 MHz, DMSO-d_6): δ = 178.72 (Acetate), 178.32 (Acetate), 177.99 (Biotin-1) 166.95 (Biotin-10), 64.45 (Biotin-8), 61.63 (Biotin-9,DACH-1), 59.58 (DACH-1), 53.60 (Biotin-6), 41.02 (Biotin-7), 37.17 (Biotin-2), 31.26 (DACH-2), 30.83 (DACH-2), 27.19 (Biotin-5), 27.15 (Biotin-4), 24.46 (Biotin-3), 23.84 (DACH-3), 23.64 (DACH-3), 23.30 (Acetate), 23.10 (Acetate) ppm; MS:

calcd. for [$C_{20}H_{36}N_4O_8PtS$-H^+]$^+$: 688.1975, found: 688.1973; elemental analysis calcd. for $C_{20}H_{36}N_4O_8PtS \cdot 1.5\ H_2O$: C: 33.61, H: 5.50, N: 7.84, S: 4.49, found: C: 33.50, H: 5.17, N: 8.07, S: 4.42.

Characterization of BisEqBio:

^{1}H-NMR (500 MHz, DMSO-d$_6$): δ = 8.84 (m, 2H, N*H*$_2$), 8.15 (b, 2H, N*H*$_2$), 6.41 (b, 2H, N*H*), 6.35 (b, 2H, N*H*), 4.31 (m, 2H, Biotin-8), 4.13 (m, 2H, Biotin-9), 3.08 (m, 2H, Biotin-6), 2.82 (dd, 2H, Biotin-7, *J* = 12.43 Hz, *J* = 5.07 Hz), 2.58 (b, 2H, Biotin-7), 2.52 (b, 2H, DACH-1), 2.27 (m, 4H, Biotin-2), 2.21 (m, 2H, DACH-2), 1.90 (s, 6H, Acetate), 1.63-1.58 (m, 2H, Biotin-4), 1.53-1.43 (m, 8H, DACH-3,Biotin-3,Biotin-4), 1.39-1.28 (m, 6H, DACH-2,Biotin-3/Biotin-4), 1.09 (m, 2H, DACH-3) ppm. ^{13}C-NMR (125 MHz, DMSO-*d*$_6$): δ = 179.49 (Acetate), 178.27 (Biotin-1), 162.74 (Biotin-10), 61.38 (DACH-1), 61.09 (Biotin-9), 59.19 (Biotin-8), 55.59 (Biotin-6), 39.62 (under the solvent peak, Biotin-7), 35.01 (Biotin-2), 30.56 (DACH-2), 28.19 (Biotin-4,Biotin-5), 25.88 (Biotin-3), 23.66 (DACH-3), 22.98 (Acetate) ppm; MS: calcd. for [$C_{30}H_{50}N_6O_{10}PtS_2$-$Na^+$]$^+$: 936.2572, found: 936.2570; elemental analysis calcd. for $C_{30}H_{50}N_6O_{10}PtS_2 \cdot 2.5\ H_2O$: C: 37.57, H: 5.78, N: 8.76, S: 6.69, found: C: 37.30, H: 5.44, N: 8.80, S: 6.65.

BisEqMalEs

(OC-6-33)-Diacetato[(1*R*,2*R*)-cyclohexane-1,2-diamine]di[6-(2,5-dioxo-2,5-dihydro-1*H*-pyrrol-1-yl)hexanoato]platinum(IV)

Purified [(DACH)Pt$(OH)_2(OAc)_2$] (100 mg, 0.217 mmol) was suspended in 4 ml DMF, 6-(2,5-dioxo-2,5-dihydro-1H-pyrrol-1-yl)hexanoic acid (275 mg, 1.30 mmol; 6 eq.) was added and the mixture was stirred for 24 h at 50°C. The solvent was removed in vacuo and the crude product was purified via preparative RP-HPLC with an isocratic gradient of 32% ACN (solvents without added TFA) Yield: 75 mg (41%) as a white solid. ^{1}H-NMR (700 MHz, DMSO-d$_6$): δ = 8.87 (b, 2H, N*H*$_2$), 8.15 (b, 2H, N*H*$_2$), 6.99 (s, 4H, MalEs-8), 3.36 (t, 4H, MalEs-6, *J* = 7.19 Hz), 2.58 (b, 2H, DACH-1), 2.23 (m, 6H, DACH-2,MalEs-2), 1.90 (s, 6H, Acetate), 1.50-1.44 (m, 10H, DACH-3,MalEs-3,MalEs-5), 1.34 (b, 2H, DACH-2), 1.23 (m, 4H, MalEs-4, *J* = 7.60 Hz), 1.09 (t, 2H, DACH-3, *J* = 9.59 Hz) ppm. ^{13}C-NMR (175 MHz, DMSO-*d*$_6$): δ = 179.47 (Acetate), 178.15 (MalEs-7), 171.05 (MalEs-1), 134.43 (MalEs-8), 61.37 (DACH-1), 37.01 (MalEs-6), 34.99 (MalEs-2), 30.57 (DACH-2), 27.83 (MalEs-5), 25.63 (MalEs-4), 25.28 (MalEs-3), 23.65 (DACH-3), 22.92 (Acetate) ppm; MS: calcd. for [$C_{30}H_{44}N_4O_{12}Pt$-Na^+]$^+$: 870.2496, found: 870.2485; elemental analysis calcd. for $C_{30}H_{44}N_4O_{12}Pt \cdot 1\ H_2O$: C: 41.62, H: 5.36, N: 6.47, found: C: 41.58, H: 5.10, N: 6.86.

BisEqMalCa

(OC-6-33)-Diacetato[(1*R*,2*R*)-cyclohexane-1,2-diamine]di[5-(2,5-dioxo-2,5-dihydro-1*H*-pyrrol-1-yl)pentylcarbamato]platinum(IV)

Purified [(DACH)Pt(OH)$_2$(OAc)$_2$] (100 mg, 0.217 mmol) was suspended in 4 ml DMF, 1-(5-isocyanatopentyl)-1H-pyrrole-2,5-dione (180 mg, 0.868 mmol; 4 eq.) was added and the mixture was stirred for 24 h at room temperature. The solvent was removed in vacuo and the crude product was purified via preparative RP-HPLC with an isocratic gradient of 26% ACN (solvents without added TFA) Yield: 77 mg (40%) as a white solid. ^{1}H-NMR (500 MHz, DMSO-d$_6$): δ = 9.00 (b, 2H, N*H*$_2$), 8.28 (b, 2H, N*H*$_2$), 7.00 (s, 4H, MalCa-8), 6.20-5.70 (m, 2H, MalCa-N*H*), 3.40-3.35 (m, 4H, MalCa-6, *J* = 4,78 Hz), 2.89 (b, 4H, MalCa-2), 2.66 (b, 2H, DACH-1), 2.22-2.20 (dd, 2H, DACH-2, *J* = 11.50 Hz), 1.88 (s, 6H, Acetate), 1.51-1.43 (m, 6H, DACH-3,MalCa-5), 1.37-1.32 (m, 6H, DACH-2,MalCa-3), 1.22-1.17 (m, 4H, MalCa-4), 1.09 (t, 2H, DACH-3) ppm. ^{13}C-NMR (125 MHz, DMSO-*d*$_6$): δ = 179.93 (Acetate), 178.15 (MalCa-7), 171.06 (MalCa-1), 134.42 (MalCa-8), 61.40 (DACH-1), 40.91 (MalCa-2), 37.06 (MalCa-6), 29.54 (DACH-2), 27.74 (MalCa-5), 23.67 (DACH-3), 23.52 (MalCa-4), 23.19 (MalCa-3); MS: calcd. for $[C_{30}H_{44}N_4O_{12}Pt\text{-}Na^+]^+$: 900,2714, found: 900,2684; elemental analysis calcd. for $C_{30}H_{46}N_6O_{12}Pt$: C: 41.05, H: 5.28, N: 9.57, found: C: 40.67, H: 5.27, N: 9.84.

Stability and reduction experiments

Phosphate buffer (100 or 150 mM, pH 7.4) containing 1 mM platinum compound was incubated for 24 h. For stability studies the complexes were incubated at 37°C, while reduction kinetics were conducted at 20°C, with the addition of 10 eq. L-ascorbic acid. The reaction was monitored on a Thermo Scientific Dionex UltiMate 3000 UHPLC-system using a Waters Acquity UPLC BEH C18 1.7 µm 3.0x50 mm column. Milli-Q water, containing 0.1% formic acid, and acetonitrile containing 0.1% formic acid were used as eluents. A gradient of 5-95% over 5 minutes was used.

SEC-ICP-MS measurements

Fetal calf serum (FCS) was purchased from Sigma-Aldrich and buffered with 150 mM phosphate pH 7.4 in order to guarantee a stable pH over the duration of the experiment. The platinum(IV) complexes (5 mM) were dissolved in 150 mM phosphate buffer (pH 7.4) and diluted 1:50 in the buffered serum to obtain a final concentration of 100 µM. The samples were then incubated in the autosampler at 37°C and analyzed every 1 h for 5 h as well as after 24 h. Between each sample a pure water blank was measured. For SEC-ICP-MS measurements an Agilent 1260 Infinity system coupled to an Agilent 7800 ICP-MS equipped with

a dynamic reaction cell was used. Oxygen (purity 5.5, Messer Austria GmbH, Gumpoldskirchen, Austria) was used as reaction gas. HPLC parameters are given in Table S1 and ICP-MS operation parameters are given in Table S2.

Biological evaluations

Cell culture

For *in vitro* experiments, the human breast cancer model MCF-7 and the colon cancer cell model HCT116 (purchased from American Type Culture Collection, Manassas, VA, USA) were used and were grown in either DEMEM or McCoy medium, respectively. For *in vivo* experiments the murine (Balb/c) CT-26 (colon cancer) tumor model was used which were cultured in DEMEM/F-12 medium. The media were supplemented with 10% FCS (purchased from PAA Linz, Austria). All cells were not treated with antibiotics and were regularly checked for mycoplasma contamination. All cell culture media and reagents were purchased from Sigma-Aldrich Austria.

Cellular biotin uptake determined by flow cytometry

The cells were plated in 12-well plates (2.5 x 10^5 cells/ml/well) and left to recover for 24 h. FITC-conjugated biotin (20 µM, 53608, Sigma Aldrich) was diluted with serum-free DEMEM medium and added to the cells. After 5 h the cells were harvested by trypsinization, diluted in phosphate-buffered saline (PBS) and the fluorescence was measured by flow cytometry (excitation: 488 nm and emission: 530 nm) using a BD Fortesssa flow cytometer. Further data processing was done by FlowJo software.

Cellular drug uptake determined by ICP-MS

The cells were plated in 6-well plates (1 x 10^6 cells /ml/well) and left to recover for 24 h. After further 24 h of serum starvation, drugs (10 µM) were added to the cells for 5 h in triplicates. Drug solution was also added to blank wells without cells in triplicates, in order to assess absorption of the compounds to the plastic of the well. The protocol by Egger et al. was followed for sample processing, measurement and data evaluation.[7] The platinum concentration was determined by ICP-MS analysis. Platinum and rhenium standards were derived from CPI International (Amsterdam, The Netherlands). The total platinum content was determined with a quadrupole-based ICP-MS instrument Agilent 7800 (Agilent Technologies, Tokyo, Japan) equipped with the Agilent SPS 4 autosampler (Agilent Technologies, Tokyo, Japan) and a MicroMist nebulizer at a sample uptake rate of approximately 0.2 ml/min. An RF power of 1550 W was used as well as nickel cones. Argon was used as plasma gas (15 l/min) and as carrier gas (~1.1 l/min). The dwell time

was set to 0.1 s and the measurements were performed in 12 replicates with 100 sweeps. Rhenium served as internal standard for platinum. The Agilent MassHunter software package (Workstation Software, version B.01.04, 2018) was used for data processing.

Cell viability assay

Cells were plated in 96-well plates (2-3 x 10^4 cells in 100 µL per well) and left to recover for 24 h. Subsequently, the cells were incubated with the indicated drugs in rising concentrations. All substances were freshly dissolved either in water (oxaliplatin) or DMF before further dilution in cell culture medium. DMF concentrations were always below 0.5%. After 72 h incubation, cell viability was analyzed using an MTT-based assay (EZ4U, Biomedica, Austria) following the manufacturer´s recommendations. The received data was visualized as dose-response curves by Graph Pad Prism 5 software (Graph Pad software, USA).

Colony formation assay

The cells were plated in 24-well plates (250 cells in 500 µl growth medium per well) and left to recover for 24 h in the incubator. Subsequently, the test compounds were added to the cells in rising concentrations in duplicates. After 10 days, the cells were fixed with MeOH for 20 min at 4°C and stained with crystal violet (0.01% in PBS). The fluorescence of crystal violet was detected by the Typhoon scanner (Typhoon TRIO Variable Mode Imager, GE Healthcare Life Sciences) after excitation by the red laser (633 nm). Quantification was done by ImageJ software. The received data was visualized as dose-response curves by Graph Pad Prism 5 software (Graph Pad software, USA).

Animals

Eight- to twelve-week-old Balb/c mice were purchased from Envigo Laboratories (San Pietro al Natisone, Italy). The animals were kept in a pathogen-free conditions, controlled environment with 12 h light–dark cycle and every procedure was done in a laminar airflow cabinet. All experiments were approved by the Ethics Committee for the Care and Use of Laboratory Animals at the Medical University Vienna (BMWF-2022-0.770.291) and performed according to the guidelines from the Austrian Animal Science Association and from the Federation of European Laboratory Animal Science Associations (FELASA).

Serum pharmacokinetics (PK) and tumor accumulation of BisEqMalEs and BisEqMalCa in CT-26-bearing mice

CT-26 cells ($5x10^5$ in 50 µl serum-free medium) were injected subcutaneously (s.c.) into the right flank of male Balb/c mice. After 10 days of cell injection, the drugs were applied i.v. (n=4 per treatment group) with a single dose equimolar to 9 mg/kg oxaliplatin dissolved in 20% propylene glycol (PG) and 5% glucose. For serum PK, blood was collected after 5 min, 30 min, 5 h and 24 h (2 mice per time point) via the facial vein. The serum was separated by centrifugation (two times 10 min at 900 g). The tumor accumulation was determined after 5 h and 24 h (2 animals per time point). Therefore, the animals were sacrificed via cervical dislocation and the tumor was harvested. All collected samples were stored at -20°C and further processed for platinum measurements via ICP-MS. HNO_3 (67-69%, suprapur for trace metal analysis, NORMATOM; Distributor: VWR international, Austria) and conc. H_2O_2 suprapur (Merck, Wasserstoffperoxid 30%) were used without further purification. Digestion of tissue (approx. 15-30 mg gravimetrically weighted) was performed with 2 ml of approx. 20% nitric acid and 100 µl H_2O_2 using an open vessel graphite digestion system (coated graphite heating plate, coated sample holder-top for 25 ml vials, PFA vials and PFA lids; Labter, ODLAB; Distributor: AHF Analysentechnik AG; Germany). Digested samples were diluted in Milli-Q water (18.2 MΩ cm, Milli-Q Advantage, Darmstadt, Germany). The platinum concentration was determined by ICP-MS analysis using the same instrumental conditions as for platinum analysis in cells.

Anticancer activity experiment *in vivo*

CT-26 cells ($5x10^5$ in serum-free medium) were injected s.c. into the right flank of male Balb/c mice. Therapy was started when tumor nodules were palpable (day 4). Animals were treated with **BisEqMalEs** (19.6 mg/kg i.v. dissolved in 20% PG and 5% glucose) and **BisEqMalCa**, (19.9 mg/kg i.v. dissolved in 20% PG and 5% glucose) and the equimolar dose of oxaliplatin (9 mg/kg i.v. dissolved in 5% glucose) on day 4, 7, 11 and 14 after cell injection. Animals were controlled for distress development every day and tumor size was assessed regularly by caliper measurement. Tumor volume was calculated using the formula: (length × width^2)/2. In the overall survival experiments, mice were sacrificed by cervical dislocation in the case of a tumor length > 20 mm, tumor ulceration or body weight loss > 20%.

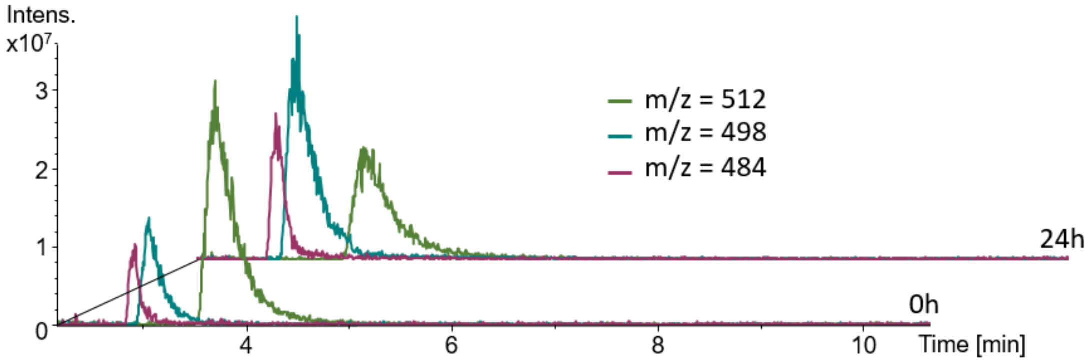

Figure S2. Hydrolysis of the MeOH complexes **1** (m/z = 498) and **2** (m/z = 512) in 50 mM phosphate buffer (pH = 7.4) at 37°C measured with HPLC-MS. m/z = 484 represents (DACH)Pt(OH_{eq})$_2$(OAc_{ax})$_2$.

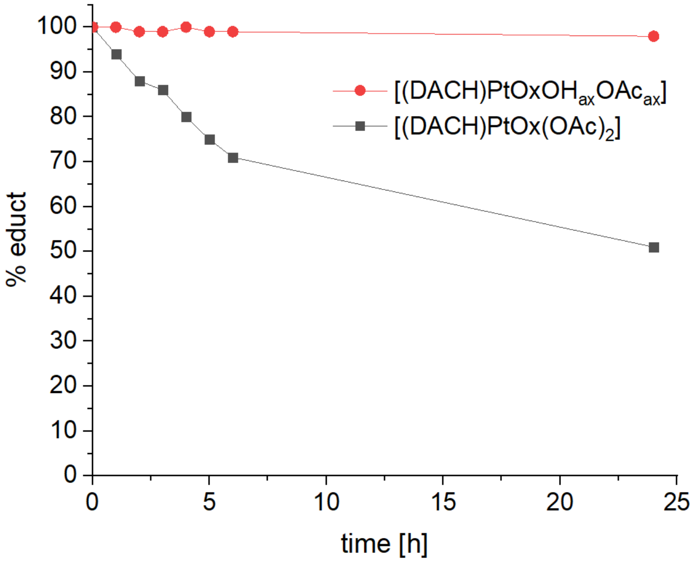

Figure S3. Equatorial hydrolysis of 1 mM [(DACH)PtOxOH$_{ax}$OAc$_{ax}$] and [(DACH)PtOx(OAc)$_2$] in 100 mM phosphate buffer 7.4 at 37°C for 24 h.

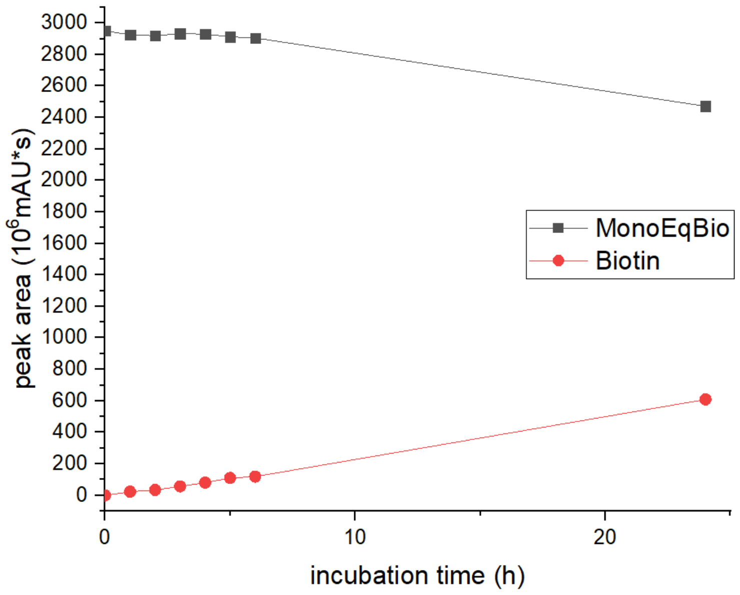

Figure S4. Equatorial hydrolysis of 1 mM **MonoEqBio** in 100 mM phosphate buffer 7.4 at 37°C for 24 h.

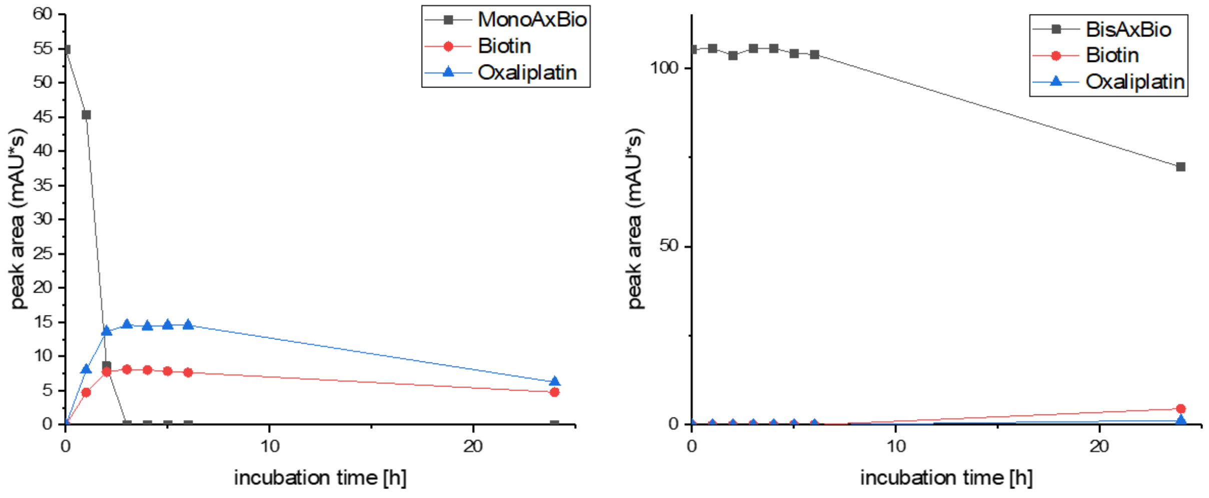

Figure S5. Reduction kinetics of **MonoAxBio** (left) and **BisAxBio** (right) at 20°C (1 mM complex in 150 mM phosphate buffer (pH 7.4) with 10 eq. of ascorbic acid) also showing the increase of biotin and oxaliplatin over time.

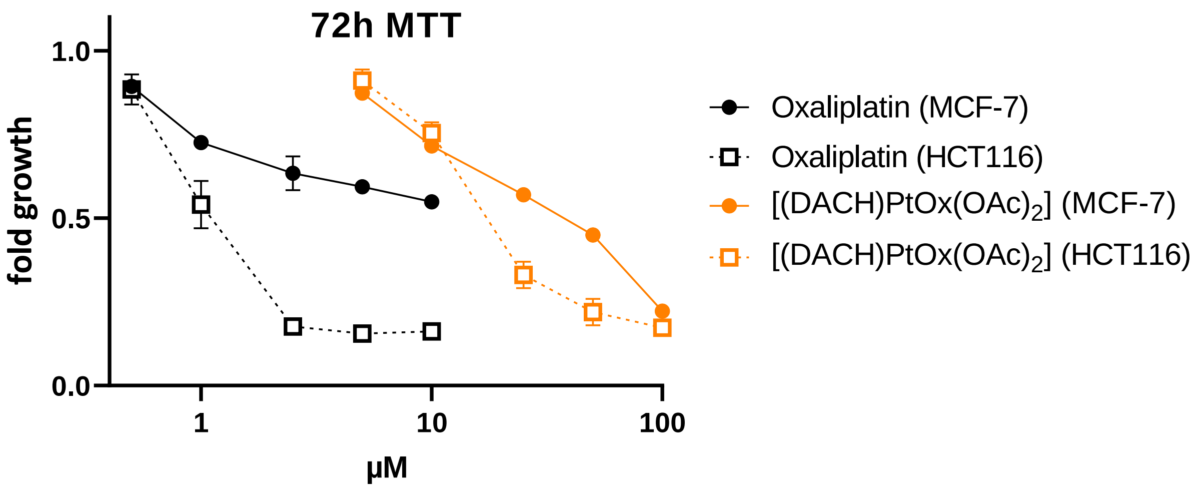

Figure S6. Anticancer activity of oxaliplatin and [(DACH)PtOx(OAc)$_2$] in MCF-7 and HCT116 cells after 72 h was determined via an MTT-based assay. Values refer to means ±SD of one representative experiment performed in triplicates.

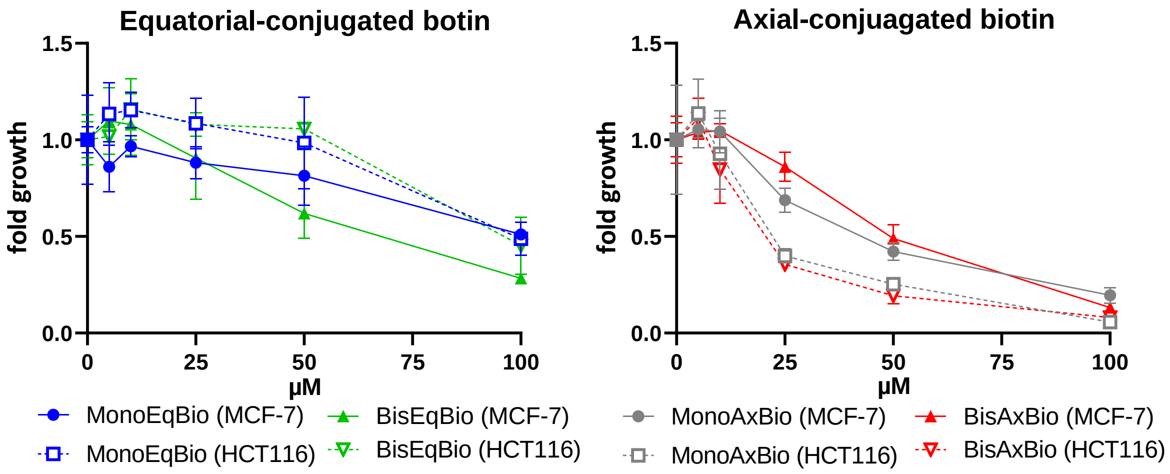

Figure S7. Anticancer activity of the biotin-conjugated platinum(IV) drugs in MCF-7 (SMVT high) and HCT116 (SMVT low) cells after long-term exposure (10 days) was determined via colony formation assays. Values refer to means ±SD of one representative experiment performed in duplicates.

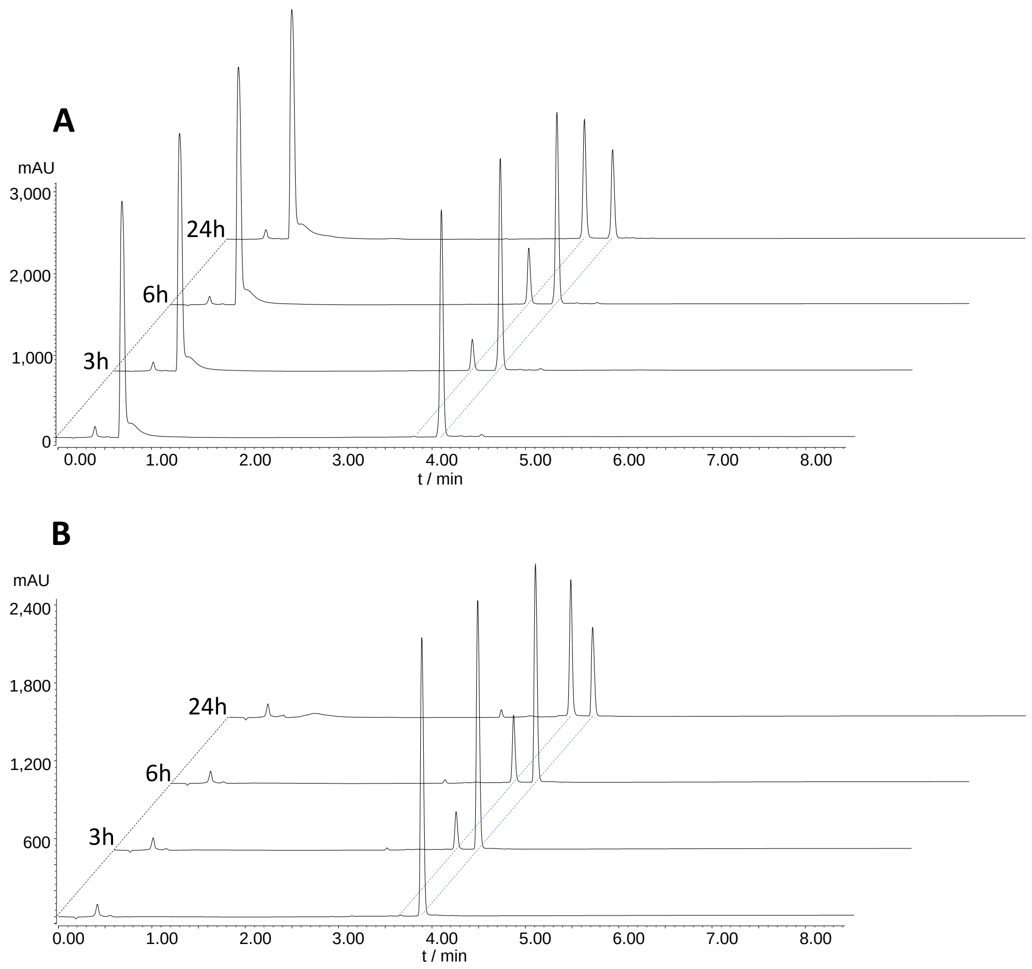

Figure S8. Stability of **BisEqMalEs** (A) and **BisEqMalCa** (B) at 20°C (1 mM complex in 150 mM phosphate buffer pH = 7.4) at 0 h, 3 h, 6 h, 24 h measured with HPLC. In both cases, the formed peak is the intact platinum(IV) complex with one hydrolyzed maleimide moiety (see Figure S9).

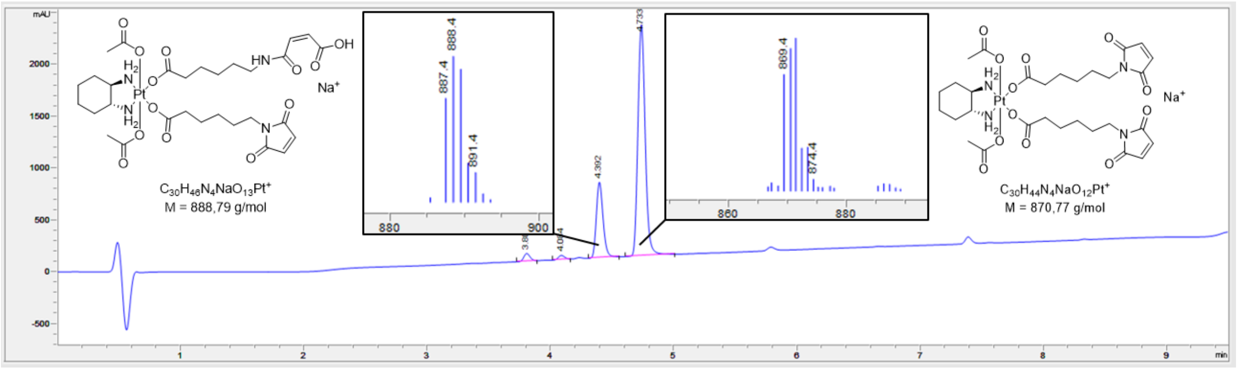

Figure S9. Peak assignment of the stability experiment depicted in Figure S8 (6 h time point of **BisEqMalEs**).

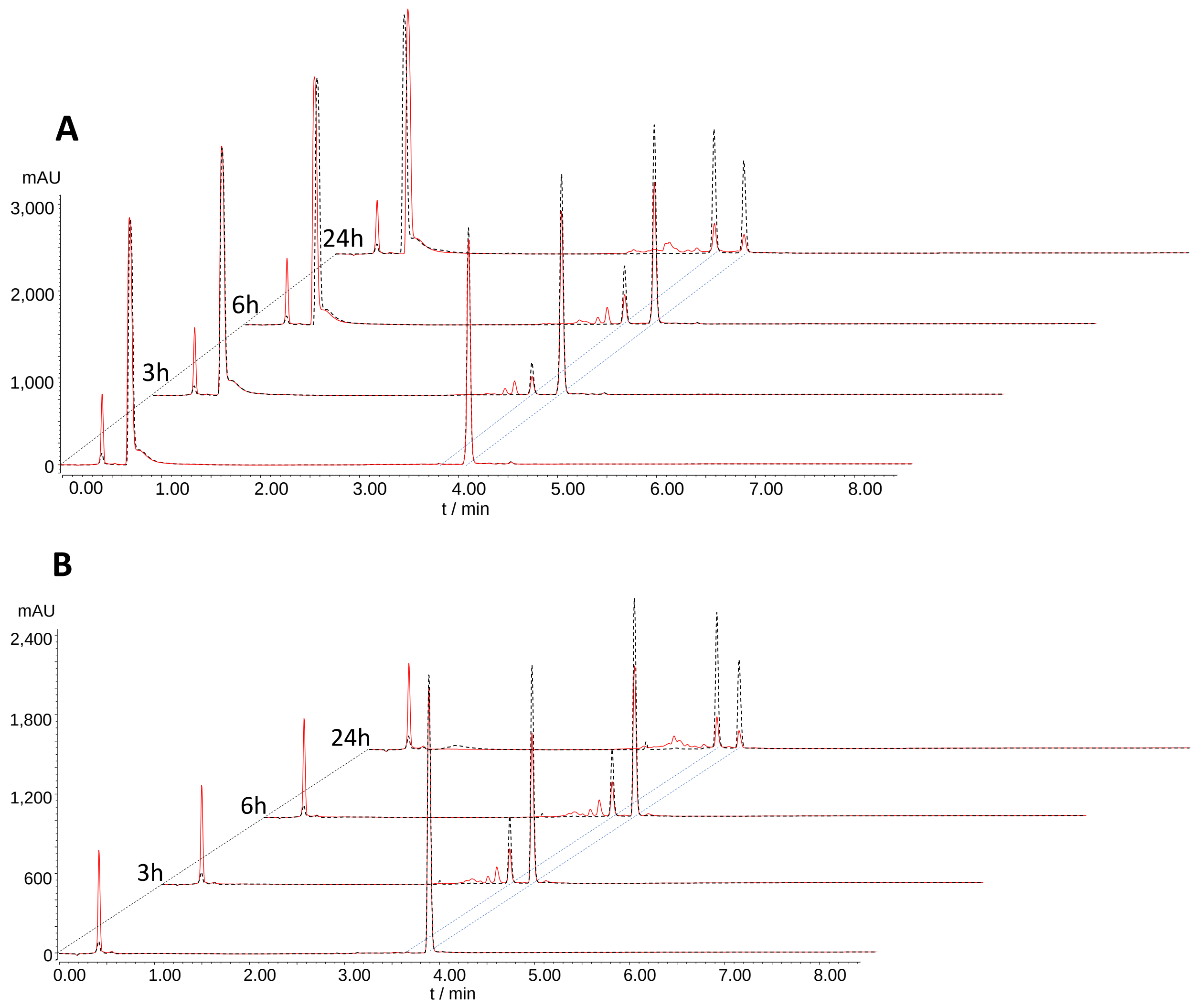

Figure S10. Comparison of stability data of (A) **BisEqMalEs** and (B) **BisEqMalCa** from Figure S7 (black dotted lines; 1 mM complex in 150 mM phosphate buffer pH = 7.4) with the respective reduction kinetics in the presence of 10 eq. of ascorbic acid (red lines; 1 mM complex in 150 mM phosphate buffer pH = 7.4) at 20°C at 0 h, 3 h, 6 h, 24 h measured with HPLC. The differences in the signal intensities of the black dotted lines and red lines of the educts and the hydrolyzed maleimide species correspond to the platinum(IV) reduction.

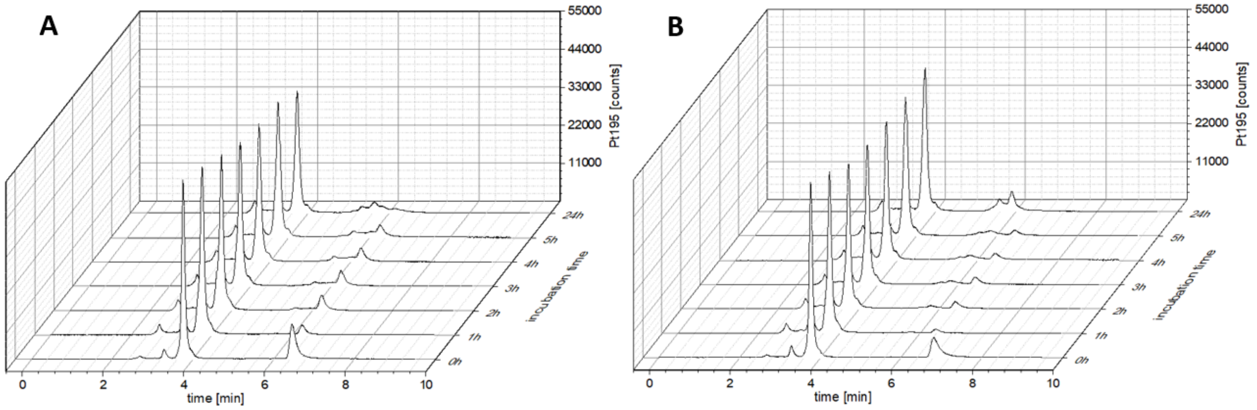

Figure S11. ^{195}Pt-traces of albumin-binding studies: incubation of 100 µM **BisEqMalEs** (**A**) and **BisEqMalCa** (**B**) in FCS (containing 150 mM phosphate buffer, pH =7.4) at 37°C over 24 h, measured with SEC-ICP-MS. Albumin-bound platinum complexes elute between 3-4 min, while free platinum species elute after 5 min.[8]

HPLC column:	Acquity UPLC BEH 200Å 1.7 µm, 4.6x150 mm
Eluent:	50 mM CH_3COONH_4, pH = 6.8
Flow rate:	400 µl/min
Injection volume:	0.5 µl
Column temperature:	37°C
Autosampler temperature:	37°C

Table S1. SEC-HPLC parameters for SEC-ICP-MS measurements.

Nebulizer:	Quartz
Spray chamber:	Scott type
Nebulizer gas flow:	1.08 l/min
Aux. gas flow:	0.9 l/min
Plasma gas flow:	15 l/min
Reaction gas (oxygen):	30 %
ICP RF power:	1550 W
m/z measured:	195, 48

Table S2. ICP-MS parameters for SEC-ICP-MS measurements.

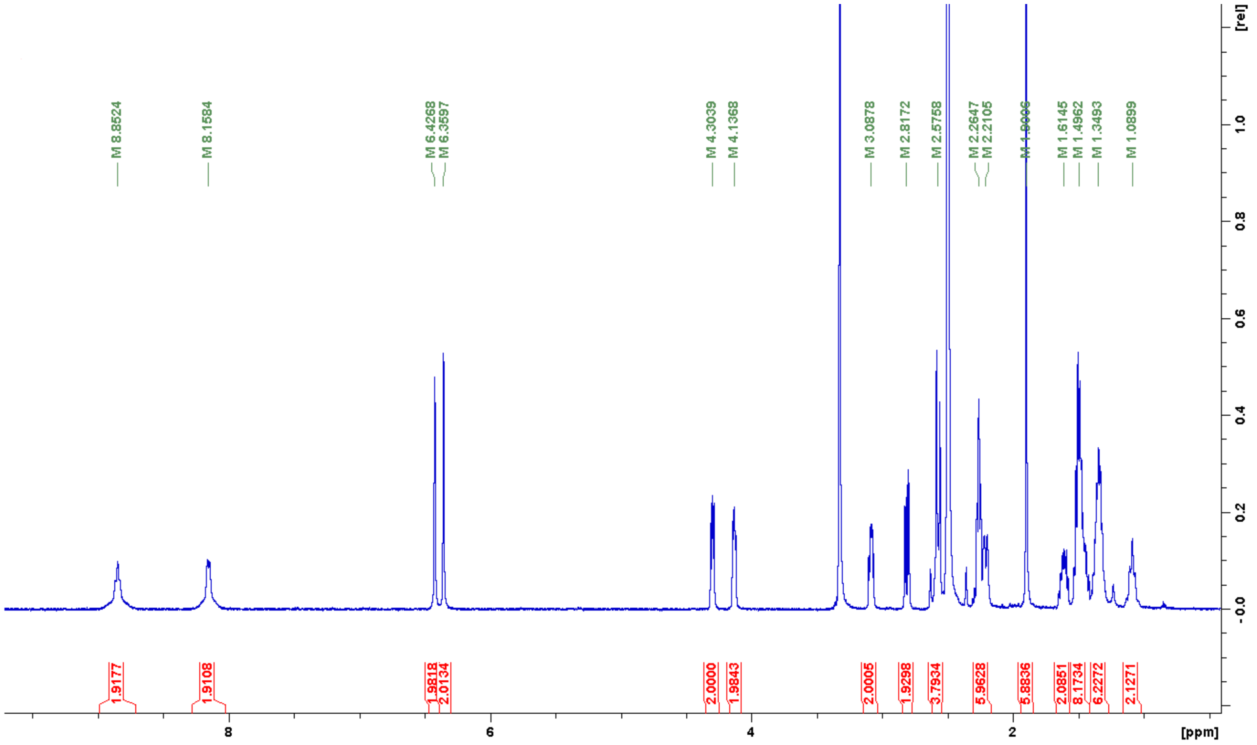

Figure S12. ^{1}H-NMR of **BisEqBio**.

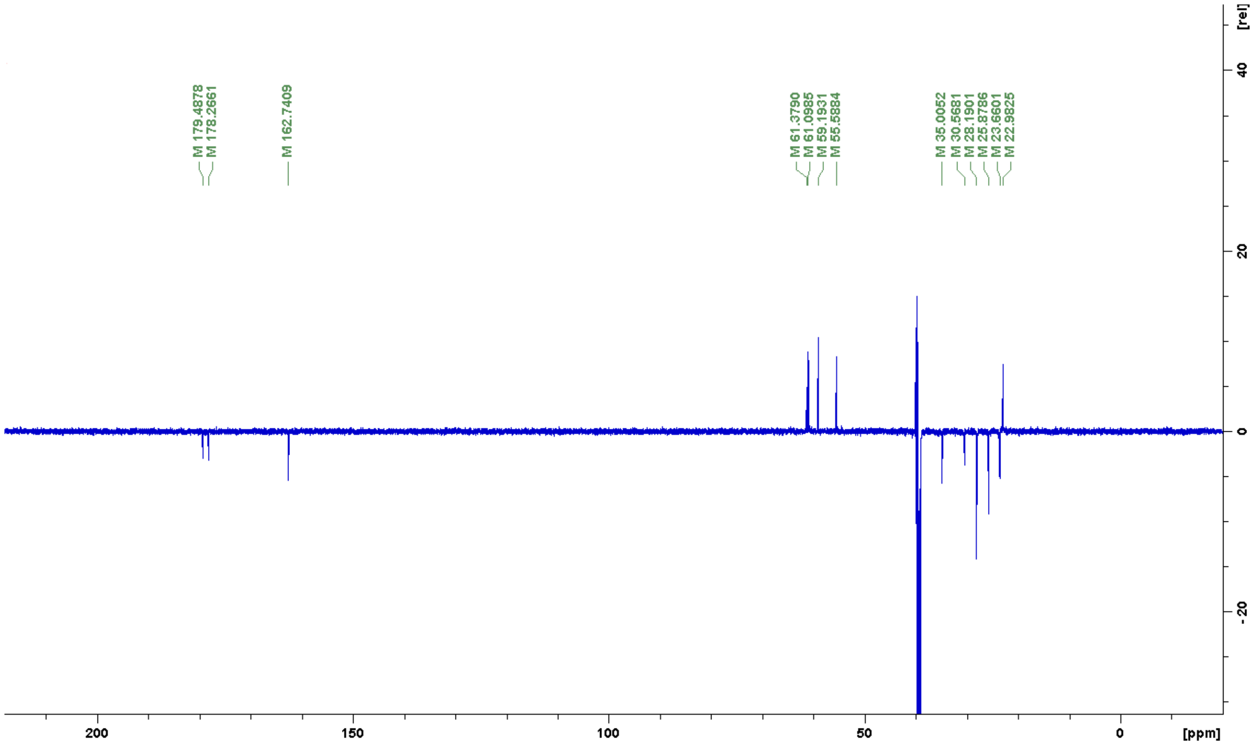

Figure S13. ^{13}C-NMR of **BisEqBio**.

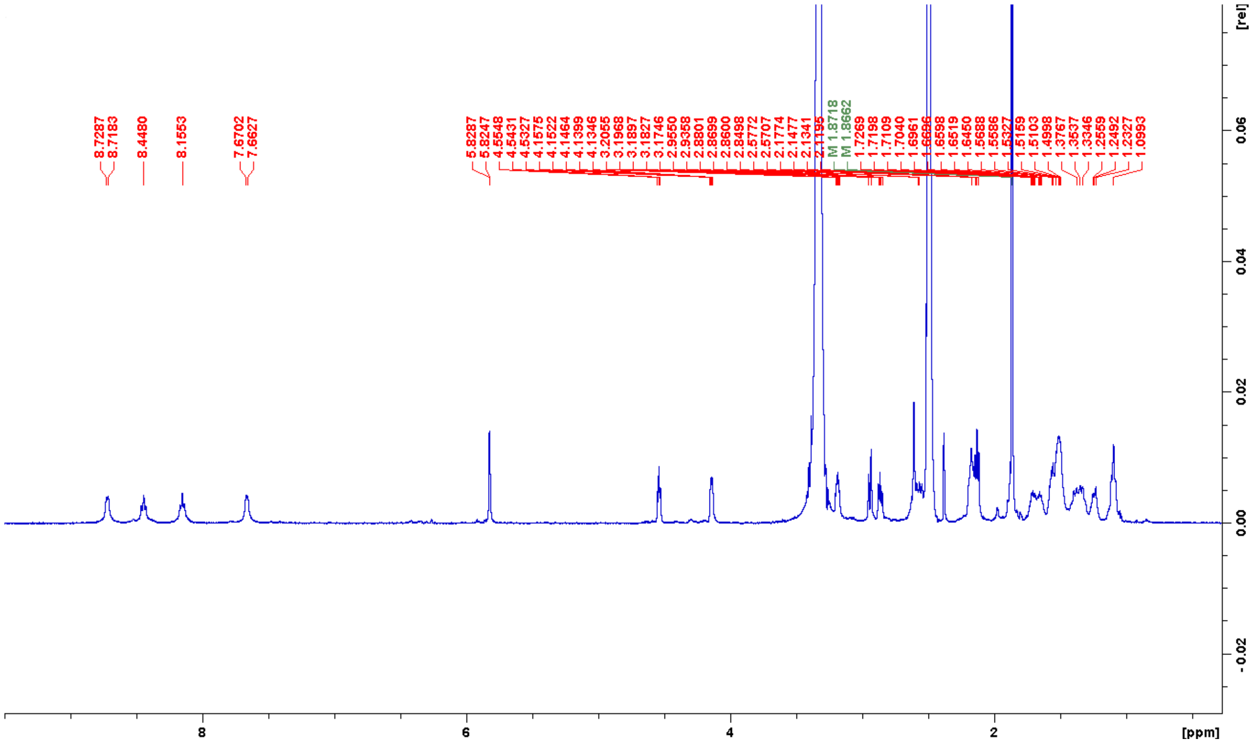

Figure S14. ^{1}H-NMR of **MonoEqBio**.

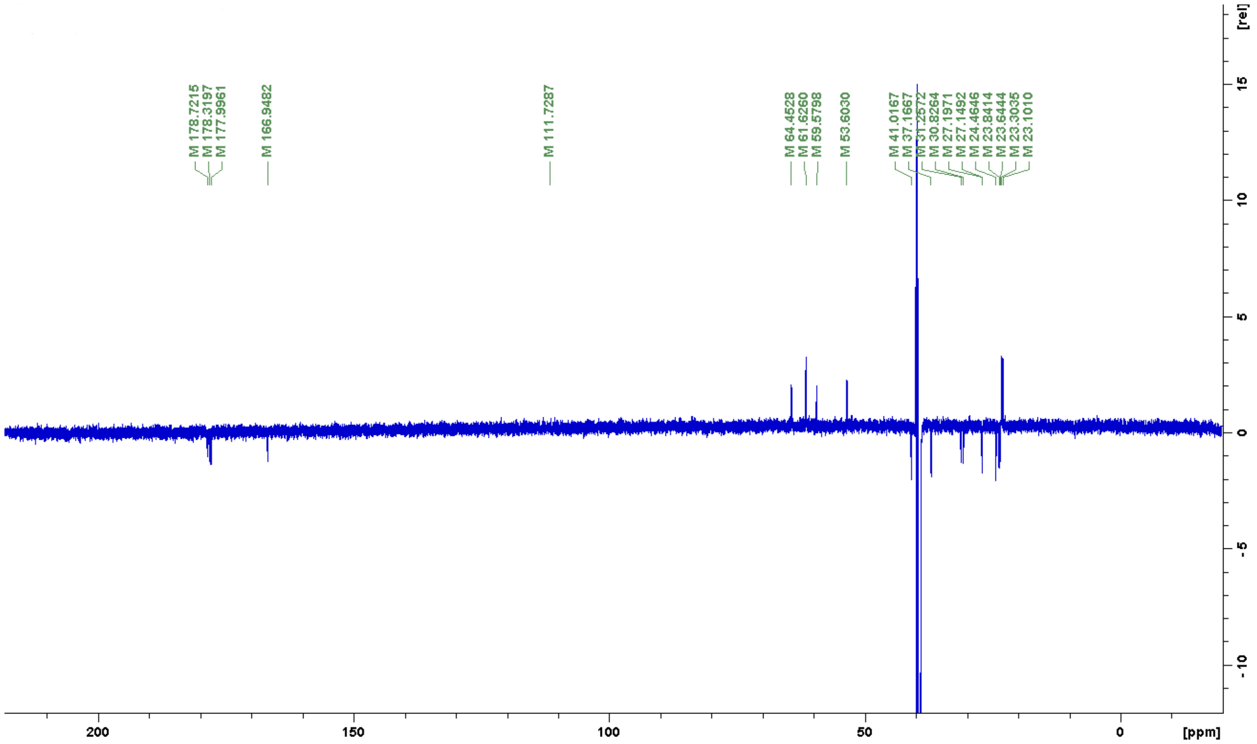

Figure S15. ^{13}C-NMR of **MonoEqBio**.

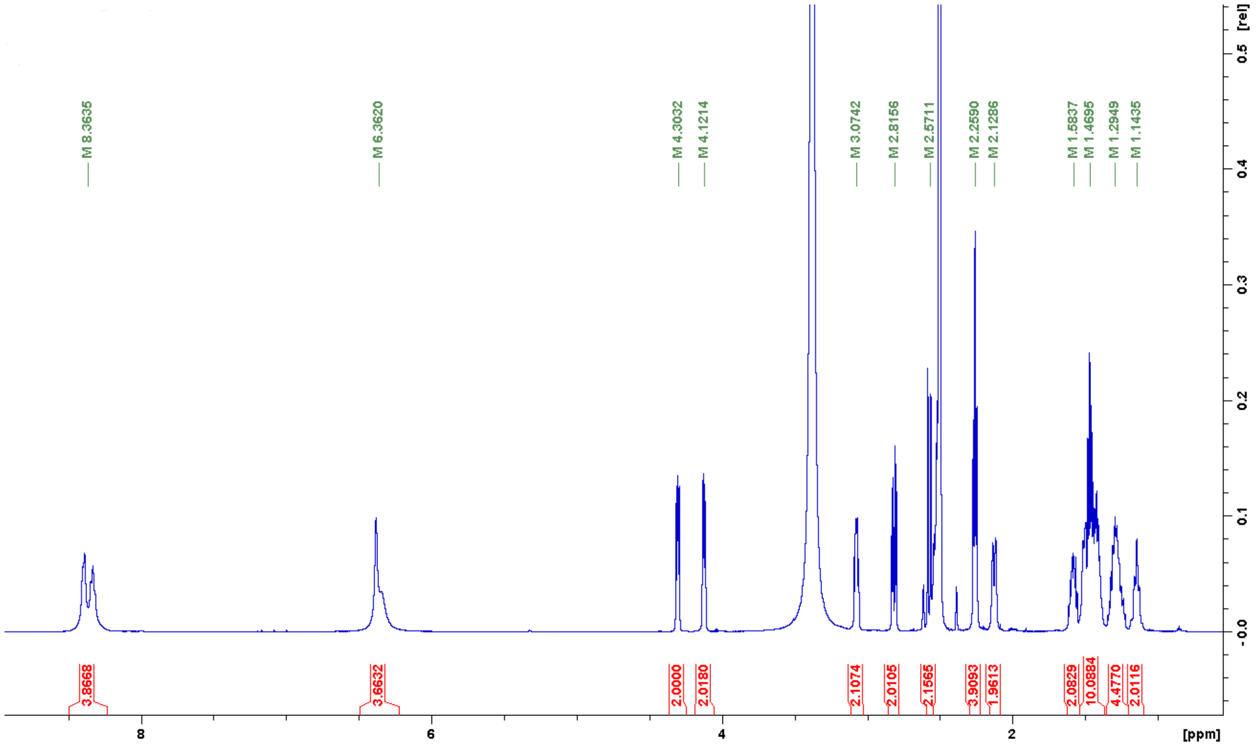

Figure S16. ^{1}H-NMR of **BisAxBio**.

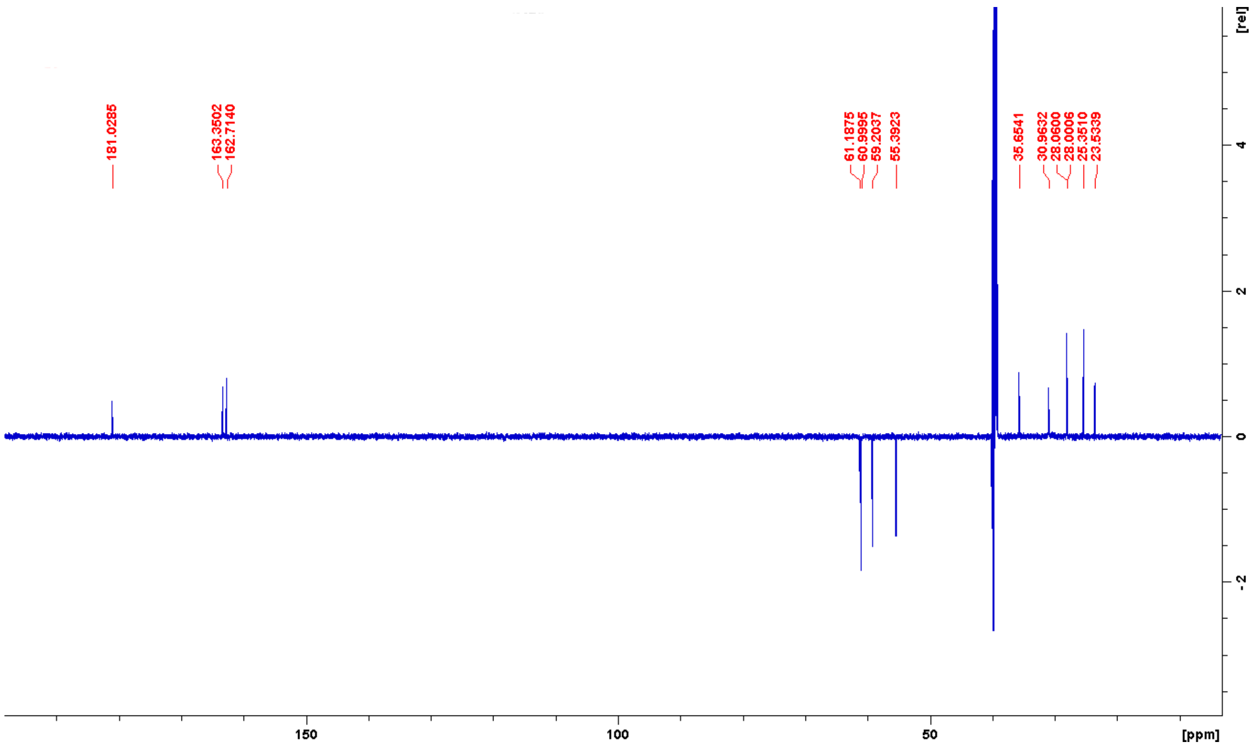

Figure S17. ^{13}C-NMR of **BisAxBio**.

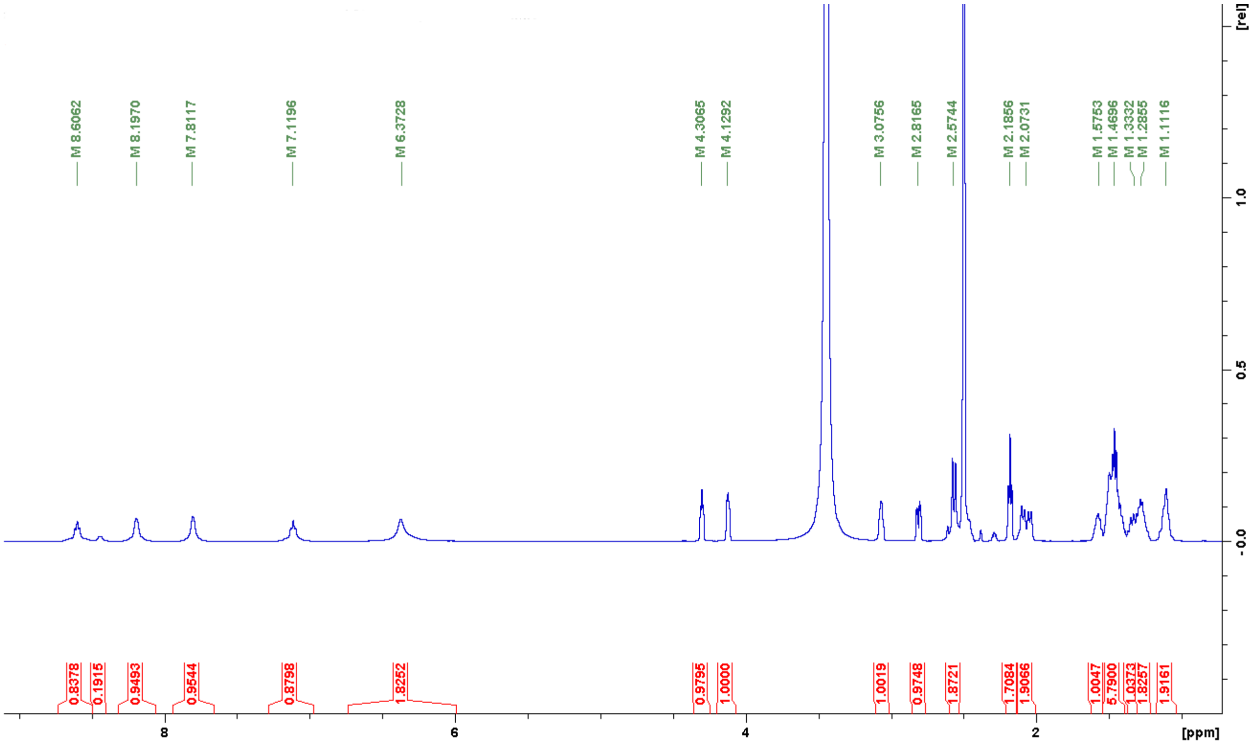

Figure S18. ^{1}H-NMR of **MonoAxBio**.

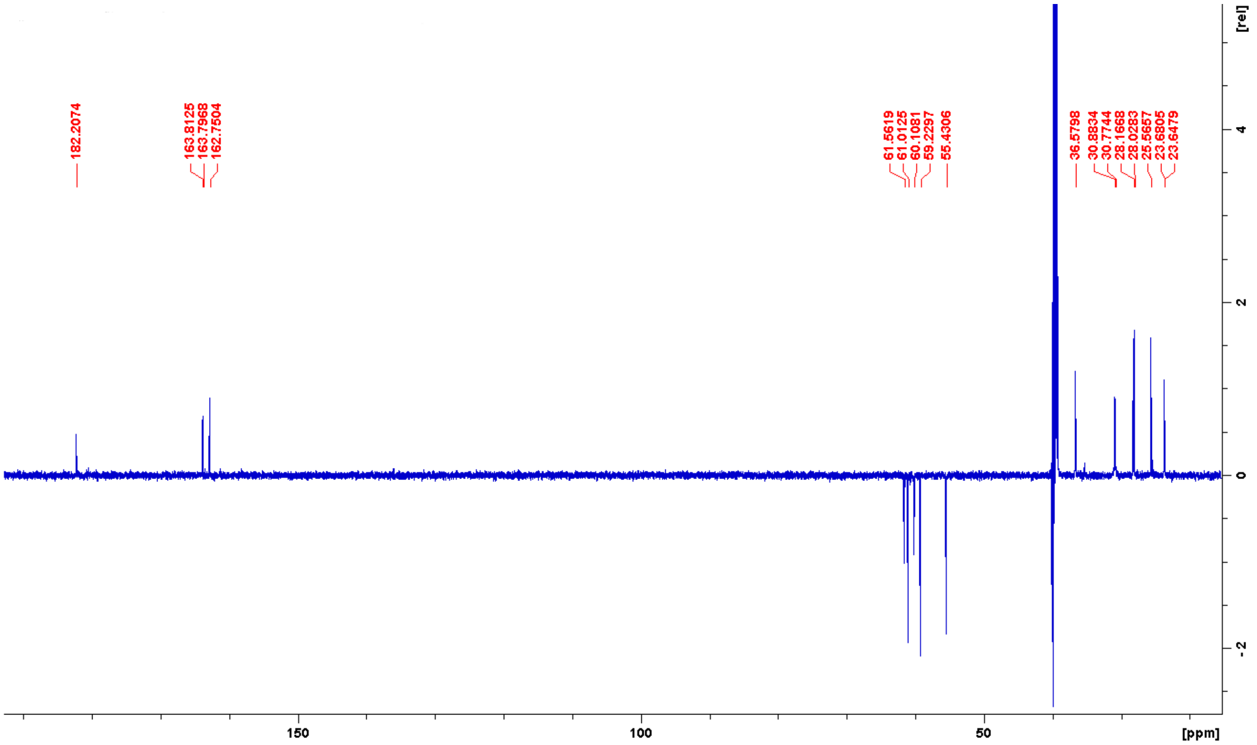

Figure S19. ^{13}C-NMR of **MonoAxBio**.

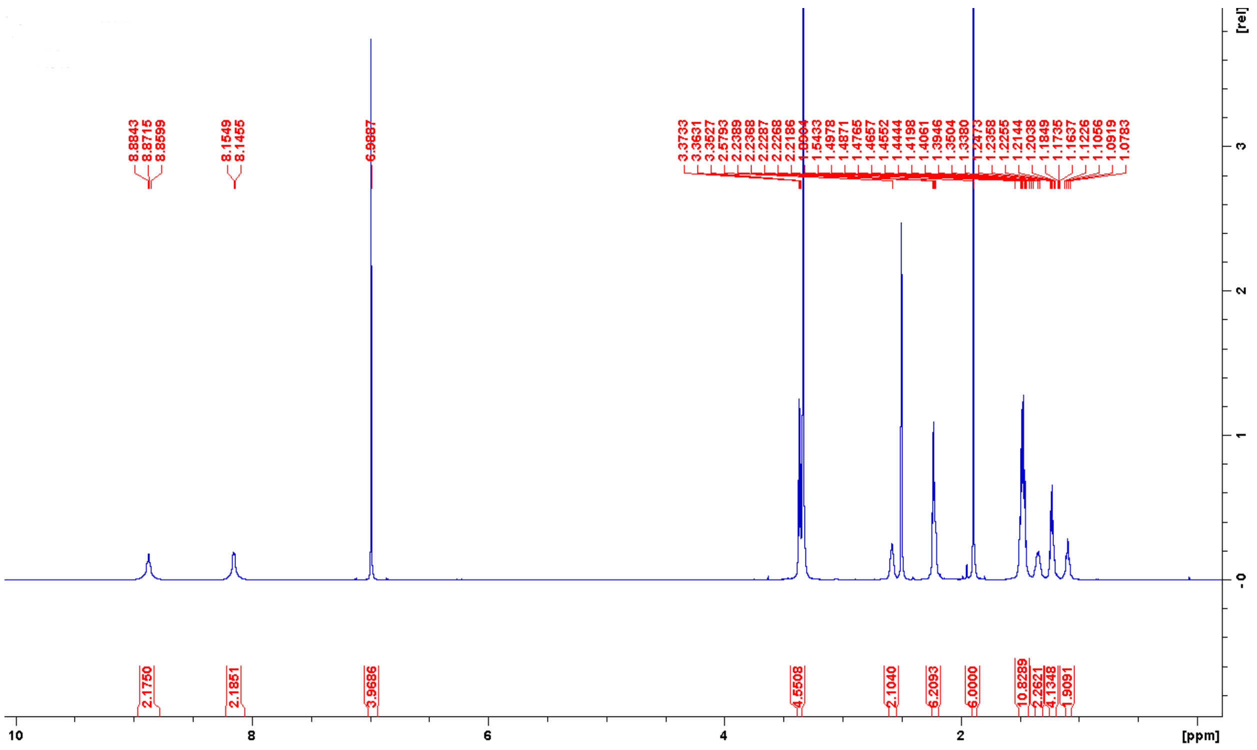

Figure S20. ^{1}H-NMR of **BisEqMalEs**.

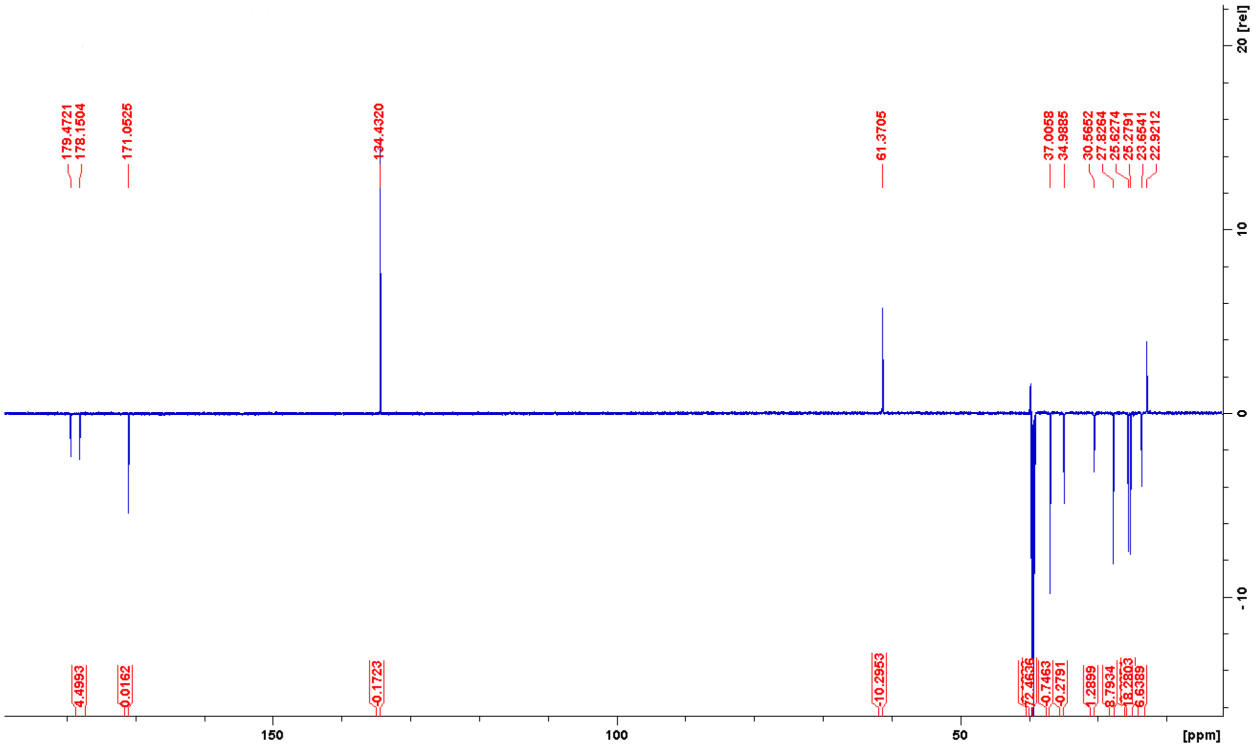

Figure S21. ^{13}C-NMR of **BisEqMalEs**.

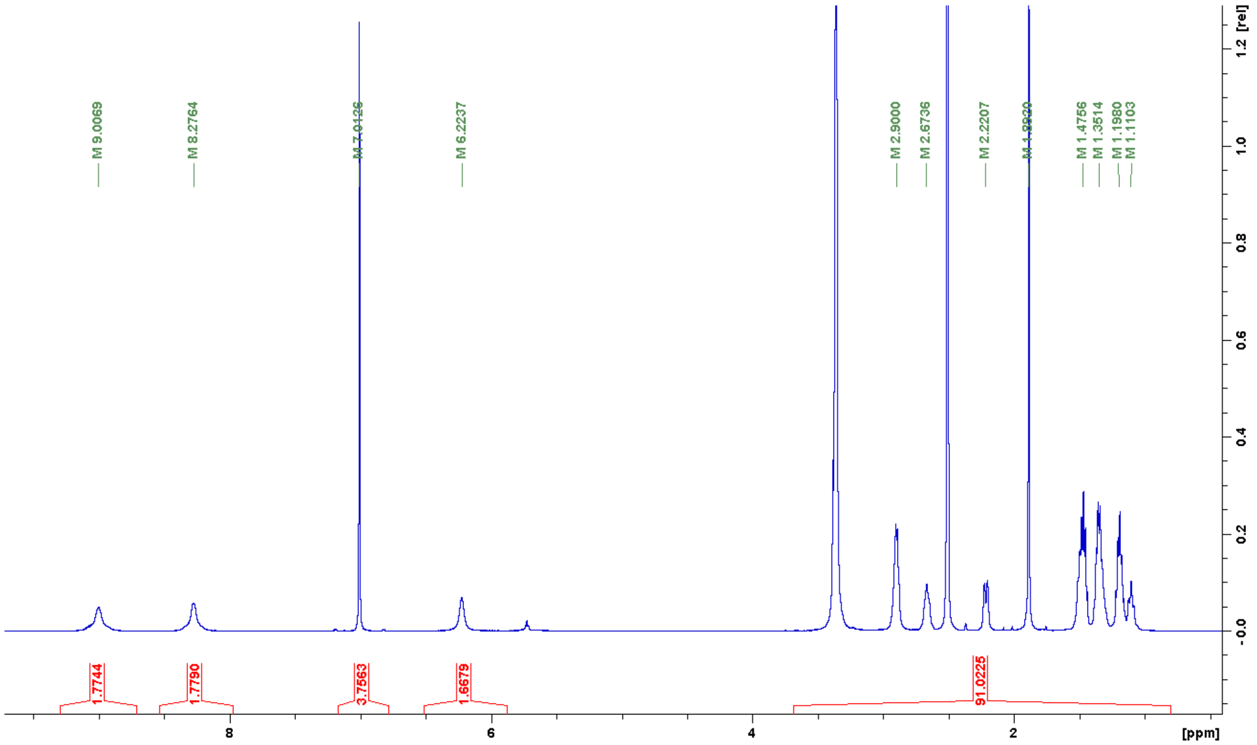

Figure S22. ^{1}H-NMR of **BisEqMalCa**.

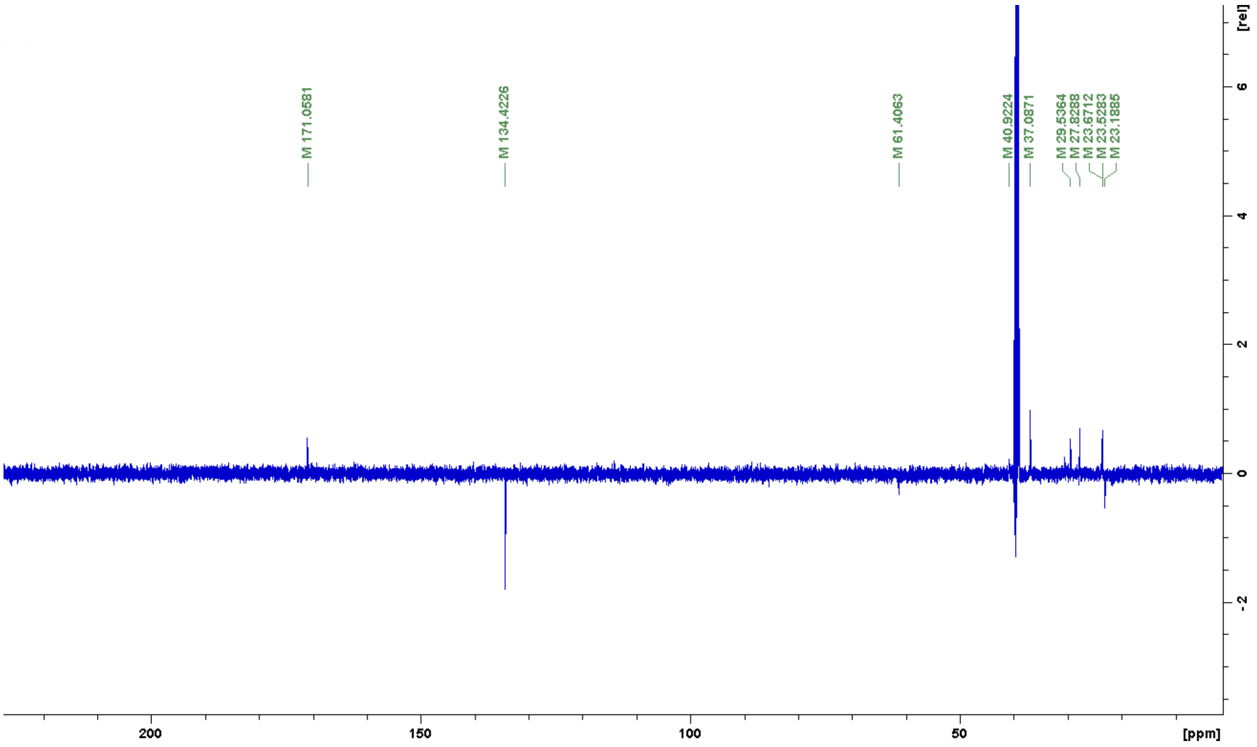

Figure S23. ^{13}C-NMR of **BisEqMalCa**.

[1] J. Z. Zhang, P. Bonnitcha, E. Wexselblatt, A. V. Klein, Y. Najajreh, D. Gibson, T. W. Hambley, *Chem Eur J* **2013**, *19*, 1672.
[2] A. Kastner, I. Poetsch, J. Mayr, J. V. Burda, A. Roller, P. Heffeter, B. K. Keppler, C. R. Kowol, *Angew Chem, Int Ed* **2019**, *58*, 7464.
[3] A. R. Khokhar, S. Al-Baker, S. Shamsuddin, Z. H. Siddik, *J Med Chem* **1997**, *40*, 112.
[4] Y.-a. Lee, S. Myung jung, S. Won kang, O.-s. Jung, *Transition Met Chem* **2004**, *29*, 710.
[5] N. Muhammad, N. Sadia, C. Zhu, C. Luo, Z. Guo, X. Wang, *Chem Commun* **2017**, *53*, 9971.
[6] P. Fronik, I. Poetsch, A. Kastner, T. Mendrina, S. Hager, K. Hohenwallner, H. Schueffl, D. Herndler-Brandstetter, G. Koellensperger, E. Rampler, J. Kopecka, C. Riganti, W. Berger, B. K. Keppler, P. Heffeter, C. R. Kowol, *J Med Chem* **2021**, *64*, 12132.
[7] A. E. Egger, C. Rappel, M. A. Jakupec, C. G. Hartinger, P. Heffeter, B. K. Keppler, *J Anal At Spectrom* **2009**, *24*, 51.
[8] A. Kastner, T. Mendrina, F. Bachmann, W. Berger, B. K. Keppler, P. Heffeter, C. R. Kowol, *Inorg Chem Front* **2023**, *10*, 4126.
